# Lactoferrin blocks orthopoxvirus entry via heparan sulphate and regulates host antiviral pathways

**DOI:** 10.1080/22221751.2026.2631205

**Published:** 2026-03-06

**Authors:** Lili Tian, Hongbo Qin, Sha Li, Mengjie Zhang, Lu Zhuang, Bixia Hong, Ke Liu, Maochen Li, Siyue Li, Yaxin Wang, Lihua Song, Yang Liu, Yun Wang, Huiyu Liu, Yigang Tong, Huahao Fan

**Affiliations:** a College of Life Science and Technology, Beijing University of Chemical Technology, Beijing, People’s Republic of China; b School of Life Sciences, Tianjin University, Tianjin, People’s Republic of China; c Wuhan Institute of Virology, Chinese Academy of Sciences, Wuhan, People’s Republic of China; d Institute of Infectious Diseases, Shenzhen Bay Laboratory, Shenzhen, People’s Republic of China; e Department of Pediatric Medicine, the Seventh Medical Center of Chinese PLA General Hospital, Beijing, People’s Republic of China; f National Engineering Laboratory for Birth Defects Prevention and Control of Key Technology, Beijing, People’s Republic of China

**Keywords:** Monkeypox, orthopoxvirus, breast milk, lactoferrin, antiviral therapy, heparan sulphate

## Abstract

Current antiviral therapies for orthopoxviruses face critical challenges, including limited efficacy and significant toxicity, which impede outbreak containment and clinical management. Here, we identify lactoferrin as a potent antiviral agent against multiple orthopoxvirus strains. Combination administration of lactoferrin with brincidofovir or tecovirimat demonstrated additive efficacy, suggesting a potential clinical strategy to reduce individual drug toxicity. Mechanistically, lactoferrin blocks viral entry by competitively binding to heparan sulphate proteoglycans (HSPGs). It also suppresses viral replication by regulating host antiviral pathways, including down-regulating cytokines and upregulating TGF-β-dependent antiviral signalling pathways. We identify TGFBI as a virus-responsive target regulated by lactoferrin. Lactoferrin treatment restores TGFBI expression and activates downstream MAPK/ERK and JAK2/STAT3 signalling cascades, leading to enhanced interferon production and interferon-stimulated gene (ISG) expression. In a murine vaccinia virus (VACV) infection model, lactoferrin treatment reduced lung viral loads and histological damage. These results underscore lactoferrin's distinctive dual antiviral mechanism and highlight its translational potential as a safe and cost-effective prophylactic or therapeutic agent. It is particularly beneficial for immunocompromised populations in resource-limited settings during orthopoxvirus outbreaks.

## 1. Main text introduction

The global surge of monkeypox virus infections (mpox) since 2022 has escalated into a public health crisis, with over 129,172 laboratory-confirmed cases and 263 deaths across 127 member states (https://worldhealthorg.shinyapps.io/mpx_global/). On August 14, 2024, the World Health Organization (WHO) declared mpox a Public Health Emergency of International Concern (PHEIC) for a second declaration [1,2]. MPXV comprises two genetic clades: Clade I, circulating in Central Africa, causes more severe disease with 10.6% mortality [3], while Clade II, specifically the B.1 lineage with enhanced transmissibility, drove the 2022 global outbreak [3–7]. Most cases of mpox are self-limiting, though approximately 40% require treatment and 13% hospitalization [8,9]. Clinical manifestations include fever, headache, myalgia, lymphadenopathy, and characteristic rash, with emerging complications such as myopericarditis and neurological sequelae [6,7]. The evolving epidemiology and emergence of virulent Clade I strains underscore the urgent need for accessible antiviral interventions [10,11]. Several smallpox therapeutics show anti-MPXV activity, including cidofovir (CDV), brincidofovir (BCV), and tecovirimat (TCV) [5,12–15]. However, TCV, despite FDA approval in 2022, failed to accelerate recovery in Clade I infections [16], while existing agents remain prohibitively expensive and supply-constrained in resource-limited settings. Most patients receive only supportive care, highlighting the critical need for effective, affordable antiviral therapy.

The mpox cases in pediatric exhibit higher disease severity and mortality compared to adults [17]. Breast milk provides both nutrition and protection against infectious diseases [18]. It contains multiple bioactive components, including lactoferrin, immunoglobulins, and human milk oligosaccharides, which have been demonstrated to exert broad-spectrum antiviral activity through synergistic mechanisms [19–21]. Lactoferrin (Lf), an 80 kDa iron-binding glycoprotein highly enriched in colostrum, represents a key bioactive component with multifaceted antimicrobial, immunomodulatory, and anti-inflammatory properties [22–24]. Notably, Lf exhibits broad-spectrum antiviral activity against diverse DNA and RNA viruses, including human immunodeficiency virus (HIV), adenovirus (AdV), poliovirus (PV), hepatitis B virus (HBV), herpes simplex virus (HSV), and severe acute respiratory syndrome coronavirus 2 (SARS-CoV-2) [23,25,26]. Lf exerts antiviral effects through multiple mechanisms across the viral life cycle. During viral entry, its N-terminal domain competitively binds host cell receptors such as heparan sulphate proteoglycans (HSPGs), low-density lipoprotein receptor (LDLR), and lamin, blocking viral attachment [26]. Lf also directly interacts with viral envelope proteins such as SARS-CoV-2 spike protein, thereby disrupting viral adsorption and entry [18]. Additionally, Lf inhibits viral envelope protein glycosylation and interferes with viral replication by binding nucleic acids or suppressing replication-associated enzymes [18]. These multi-target mechanisms, combined with its safety profile and cost-effectiveness, position Lf as a promising therapeutic candidate against emerging viral pathogens.

Despite its well-documented antiviral properties, comprehensive investigations into Lf's inhibitory effects against orthopoxvirus infections remain limited. Here, we identified lactoferrin as an effective antiviral agent against VACV and MPXV, exhibiting a broad-spectrum inhibitor against orthopoxvirus. Mechanistically, lactoferrin blocks viral adsorption through heparan sulphate binding and enhances innate immunity against viral infection. Further analysis indicates that lactoferrin suppresses viral replication by regulating host antiviral pathways, including down-regulating pro-inflammatory chemokines and cytokines and upregulating TGF-β-dependent antiviral signalling pathways. In a murine infection model, intraperitoneal lactoferrin administration significantly improved clinicopathological outcomes and reduced viral loads in the lungs. Furthermore, combination administration of lactoferrin with BCV and TCV yielded additive antiviral effects, suggesting a potential clinical strategy to reduce individual drug toxicity while maintaining efficacy.

## Materials and methods

### Cells and virus

BHK-21, Vero E6, HeLa, A549, Huh-7, and 293T cells were obtained from Cell Resource Center, Institute of Basic Medical Sciences (CAMS/PUMC) and cultured in Dulbecco's modified Eagle's medium (DMEM, Cat. 2491015, Gibco), supplemented with 10% heat-inactivated fetal bovine serum (FBS, Cat. P30-3306, PAN) and 1% Penicillin–Streptomycin Solution antibiotic (Cat. 15140122, Gibco) at 37°C, 5% CO_2_ incubator.

Vaccinia virus Tiantan strain (vvTT) (GenBank: AF095689.1) was kept in our lab. Vaccinia virus Western Reserve strain (vvWR) (GenBank: AY243312.1) was obtained from the Institute of Microbiology, Chinese Academy of Sciences. Monkeypox virus (IVCAS 6.9141, Clade IIb) was isolated from a patient in Wuhan (China) and maintained in Wuhan Institute of Virology. These viruses were passaged and titrated by standard plaque assay in Vero E6 cells.

All experiments involving monkeypox virus were conducted under Biosafety Level 3 (BSL-3) conditions at the Wuhan Institute of Virology, Chinese Academy of Sciences, which is fully certified and authorized for work with high-containment pathogens. The work was performed in strict compliance with national and institutional biosafety regulations and protocols.

### Materials and reagents

Bovine lactoferrin (Cat. S24749, Shanghai Yuanye) and human lactoferrin (Cat. L1294, Sigma-Aldrich) were used for antiviral assays. Primary antibodies included Anti-vaccinia virus antibody (Cat. ab87387, Abcam), Phospho-p44/42 and p44/42 MAPK (Erk1/2) Antibody (Cat. 4695, Cat. 4370, CST), Phospho-Stat3 and Stat3 Antibody (Cat. 9131, Cat. 9139, CST), GAPDH and Beta-actin antibody (Cat. 60004, Cat. 66009, Proteintech). Secondary antibodies comprised HRP-conjugated goat anti-rabbit or anti-mouse IgG (Cat. SA00001-1, Cat. SA00001-2, Proteintech), and CoraLite488-conjugated goat anti-rabbit IgG (Cat. SA00013-2, Proteintech). For immunofluorescence staining, 4’,6-diamidino-2-phenylindole (DAPI, Cat. H1399, Thermo Fisher Scientific) was employed. Lipofectamine™ 3000 (Cat. L3000075, Thermo Fisher Scientific) was used for transfection experiments, and the Luciferase Reporter Gene Assay Kit (Cat. 11401ES80, Yeasen) was utilized for reporter gene assays. Heparan sulphate-related reagents included heparin sodium (Cat. H8060, Solarbio), heparinase I, II, III (Cat. H2519, Cat. H6512, Cat. H8891, Sigma-Aldrich), and sodium chlorate (Cat. 244147, Sigma-Aldrich). Antiviral reference compounds brincidofovir (Cat. TQ0095, TargetMol) and tecovirimat (Cat. V3886, InvivoChem) were used for combination therapy studies.

### Collection and processing of breast milk samples

This study was approved by the Ethics Committee of the Affiliated Bayi Children's Hospital, PLA Army General Hospital (approval number S2023-025-01). Nipples were disinfected with 75% ethanol, and breast milk was collected using sterile pumps into sterile containers. Skim milk was obtained by centrifuging the samples at 4,000 × g for 15 min at 4°C, and the lower aqueous phase was retained for subsequent experiments. Donors provided informed consent and were confirmed negative for HBV, HCV, and HIV. Since the WHO declared smallpox eradicated globally in 1980 [27] and China discontinued routine smallpox vaccination in 1982, donors under 35 years old were selected to ensure absence of smallpox vaccination and anti-orthopoxvirus antibodies. Total protein concentration was measured using a BCA Protein Assay Kit (Cat. PC0020, Solarbio), and lactoferrin concentration was quantified using a Human LTF/Lactoferrin ELISA Kit (Cat. EH309RB, Thermo Fisher Scientific).

### Breast milk-derived bioactive constituents screening

Fifteen breast milk-derived bioactive constituents were selected for antiviral screening based on their documented presence and biological significance in human breast milk as reported in published proteomic, lipidomic, and compositional studies [28–30]. These components included 15 constituents (4 proteins: lactoferrin (Lf), whey protein concentrate (WPC), osteopontin (OPN), and milk fat globule membrane (MFGM); 8 human milk oligosaccharides (HMOs): 2'-fucosyllactose (2'-FL), 3'-fucosyllactose (3'-FL), 3'-sialyllactose (3'-SL), 6'-sialyllactose (6'-SL), fructooligosaccharides (FOS), galactooligosaccharides (GOS), lactose-N-tetraose (LNT), and lactose-N-neotetraose (LNnT); 3 vitamins: Vitamin B2 (VB2), Vitamin D2 (VD2), and Vitamin D3 (VD3)). Purified standards of each component were obtained commercially (BiosTime Inc). Each component was tested individually at 5 mg/ml across four cell lines (Vero, HeLa, Huh-7 and A549) using standardized antiviral assays.

### Dose–response activity detection

Dose–response activity was assessed as previously described [31]. Gene expression were quantified by RT-qPCR. The cell viability determined using the CellTiter-Blue® Cell Viability Assay (Cat. G8080, Promega) with fluorescence measured on a Synergy H1 Hybrid Multi-Mode Microplate Reader (BioTek) at 530-570 nm excitation and 590-620 nm emission. The half-maximal effective concentration (EC_50_), the half-maximal cytotoxic concentration (CC_50_), and 95% confidence intervals (CIs) were calculated using GraphPad Prism 8.0 by fitting dose–response data to a four-parameter logistic regression model. The selectivity index (SI) was calculated as the ratio of CC_50_ to EC_50_.

### DNA/RNA extraction, cDNA synthesis and quantitative real-time PCR

Total DNA and RNA were extracted from cells and virus samples using the VAMNE Virus DNA/RNA Extraction Kit 3.0 (Cat. RM501-01, Vazyme) and the Super FastPure Cell RNA Isolation Kit (Cat. RC102-01, Vazyme), respectively. For orthopoxviral DNA quantification, qPCR was performed directly without reverse transcription, as the viral genome is double-stranded DNA. For cellular RNA targets (GAPDH, CXCL2, CXCL3, and TGFBI), cDNA was synthesized using HiScript® II Q RT SuperMix (Cat. R223-01, Vazyme). All qPCR reactions were performed using Taq Pro Universal SYBR qPCR Master Mix (Cat. Q712-02/03, Vazyme). Gene expression levels were normalized to GAPDH and quantified using the 2^−ΔΔCt^ method. Viral inhibition rates were calculated as previously described [31]. Primer sequences are listed in Table S1.

### Viral titre assay

Infected cells were subjected to three freeze–thaw cycles, and supernatants were collected to quantify viral yields. The standard plaque assay was performed in Vero E6 cells overlaid with 1% methylcellulose for 3 days [19]. TCID_50_/ml values were calculated using the Reed–Muench method based on the dilution producing 50% positive wells.

### Immunofluorescence assay (IFA) and western blot (WB)

For IFA, cells were fixed with ice-cold methanol incubated with primary antibody overnight at 4℃, followed by secondary antibody for 1 h at room temperature. Nuclei were counterstained with DAPI, and fluorescence images were captured using a Nikon fluorescence microscope. For WB analysis, treated cells were lysed in RIPA buffer (cat. P0039, Beyotime). Proteins were separated by 10% SDS-PAGE, transferred to PVDF membranes, and blocked with 5% non-fat milk. Membranes were probed with primary antibody and secondary antibody, with β-actin and GAPDH as loading control. Protein bands were visualized using ECL substrate and quantified using ImageLab software version 6.0.1 (Bio-Rad).

### Time-of-addition experiments

The assay was performed as previously described [32]. Vero E6 cells were seeded into 48-well plates at 2 × 10^5^ cells/well and incubated overnight. Three treatment conditions were established with bLf at 2.5 mg/ml and vvTT at a multiplicity of infection (MOI) of 0.1. Full-Time: bLf was present throughout infection; Entry: bLf was added together with viral inoculum during two-hour adsorption at 37°C and removed by washing three times with ice-cold PBS (300 μl per well, Gibco); Post-Entry: bLf was added at 2 h post-infection (hpi) and maintained until harvest. At 14 hpi, virus yield was quantified by RT-qPCR, and viral protein expression was analysed by WB and IFA.

### Temperature shift assay

The experiment was performed as previously described [32]. For attachment assay, bLf was added with the viral inoculum (MOI = 10) during the adsorption step at 4°C for 1 h. Following adsorption, unbound virus was removed by washing the cells three times. For the internalization assay, bLf was added in viral adsorption cells and shifted to 37°C for 2 h. Following incubation, cells were washed again three times and treated with Proteinase K (100 µg/ml; Cat. 10409ES03, Yeasen) at room temperature for 10 min to digest surface-bound but non-internalized virus particles. The cell suspension was centrifuged at 1,000 × *g* for 5 min at 4°C. The cell pellet was collected in lysis buffer for DNA/RNA extraction.

### Time progression experiment assay

Cells were seeded in 48-well plates and infected with VACV at an MOI of 0.01. Following 2 h incubation at 37°C with 5% CO_2_ to allow viral entry, surface-bound and free virus were removed by washing three times with 300 µl pre-warmed PBS at 37°C. Subsequently, bLf (2.5 mg/ml) and TCV (100 nM) were added to the cells, respectively. At 2, 4, 6, 8, 10, 12, 14, and 16 hpi, both culture supernatants and cell lysates were collected for viral DNA quantification. Quantify the viral copies by comparing the obtained CT values to a standard curve generated with a quality control plasmid. This assay enabled the assessment of bLf's impact on post-entry viral replication kinetics.

### Attachment-blocking assays

For bLf pre-treatment assay, Vero E6 cells were pre-incubated with 2.5, 1.25, and 0.625 mg/ml bLf for 2 h at 37°C with 5% CO₂. Unbound bLf was removed by washing cells three times with pre-warmed PBS at 37°C, using 500 µl per wash in 24-well plates. Then cells were infected with vvTT at an MOI of 10 for 2 h at 37°C. Following infection, unbound virus was removed by washing three times, and cells were lysed for DNA/RNA extraction and quantification.

For heparin competition assay, Vero E6 cells were cultured in 96-well plates and infected with vvTT at an MOI of 0.1. Lactoferrin (250 µg/ml) was pre-incubated with varying concentrations of heparin sodium (HS) at different ratios (ranging from 5:1 to 1:3, lactoferrin:heparin). The mixture was added to cells simultaneously with virus, and viral inhibition was quantified as described above. Control groups included lactoferrin alone (without heparin). Additional competition assays were conducted using chondroitin sulphate (CS) and dermatan sulphate (DS) under identical experimental conditions.

For Sodium chlorate treatment, Vero E6 cells were cultured in 48-well plates and supplemented with 100, 50, 25, 12.5, and 6.25 mM sodium chlorate for 24 h prior to infection. Cells were then washed three times and infected with vvTT (MOI = 0.1) for 24 h at 37°C and then lysed for viral DNA quantification by qPCR. All experiments were performed in triplicate, and viral attachment efficiency was expressed as a percentage relative to untreated control cells.

### Differential scanning fluorimetry (DSF)

DSF was performed using Protein Thermal Shift Kit (Cat. 4461146, Thermo Fisher Scientific) as previously described with minor modifications [33]. Briefly, 100 µg/ml bLf was mixed with HS at concentrations ranging from 0.02 to 200 µg/ml in 96-well PCR plates. SYPRO Orange dye was added at a final concentration of 1× (diluted from 8× stock), and thermal denaturation was monitored under a temperature gradient from 20 to 95°C with an incremental step of 0.05°C/s. Continuously, fluorescence emission was recorded and the melting temperature (Tm) was determined as the midpoint of the transition from native to denatured protein using a Boltzmann sigmoidal model in Protein Thermal Shift Software v1.3 (Thermo Fisher). Dose–response curves were fitted using the Boltzmann sigmoidal equation in GraphPad Prism 8.0.

### RNA-sequencing processing and analysis

Four experimental groups were set up as follows: (i) HeLa cells cultured without any treatment; (ii) HeLa cells treated with 5 mg/ml bLf; (iii) HeLa cells infected with vvTT (MOI = 0.1); (iv) HeLa cells treated with bLf and subsequently infected with vvTT (MOI = 0.1). Each group comprised three biological replicates and was cultured for 48 h. Viral and genomic RNA was extracted using Trizol. The RNA samples were sequenced employing a standard Illumina protocol, and the reads were aligned to the reference genome using HISAT2. Gene read counts were determined with HTSeq, and differentially expressed genes (DEGs) were identified using the R package DESeq2, considering genes with a fold change of at least 2 and an adjusted *P*-value of less than or equal to 0.05. GO enrichment analysis of the DEGs was performed using the hypergeometric test to elucidate their biological functions.

### Dual-luciferase assays

Dual-luciferase reporter assays were performed using firefly luciferase driven by the target gene promoter and Renilla luciferase under the control of a constitutive cytomegalovirus (CMV) promoter as an internal normalization control. Cells were co-transfected with the indicated plasmids and luciferase reporter constructs for 12 h, followed by treatment with lactoferrin or viral infection for an additional 24 h. Cells were then washed twice with PBS and measured with Dual-Luciferase Reporter Gene Assay Kit (Cat. 11402ES60, Yeasen). Firefly luciferase activity was normalized to Renilla luciferase activity to control for transfection efficiency and cell viability.

### Animal experiment

The animal experiments conducted in this study were approved by the Animal Welfare Committee of Tianjin University, with the approval number TJUE-2024-053. We sourced pathogen-free female BALB/c mice (female, 6–8 weeks, n = 4/group) from Vital River and allowed them a 7-day acclimation period. The mice received treatment with intraperitoneal injections of bLf (100 mg/kg), tecovirimat (50 mg/kg), or placebo and intranasally challenged with 1 × 10^4^ PFU vvWR. Lungs were harvested at 5 days post-infection (dpi) for viral load quantification and histopathology (H&E, IHC). Our research group developed a Clinical Disease Severity Scoring System (CDSS) encompassing five parameters: weight, activity level, posture, coat condition, and mental state. The total score is calculated as the sum of these individual parameters. Lung specimens were fixed in 10% buffered formalin for histological assessment, stained with H&E, and processed in duplicate.

**Table:** 

Parameter	Score 0 (Normal)	Score 1 (Mild)	Score 2 (Severe)
Body Weight	<5% loss from baseline	5-15% loss from baseline	>15% loss from baseline
Activity Level	Normal movement and exploration	Reduced activity, less responsive to stimuli	Minimal movement, lethargic
Posture	Normal, upright posture	Slightly hunched or abnormal gait	Severely hunched, difficulty moving
Coat Condition	Smooth, well-groomed	Slightly ruffled or unkempt	Severely ruffled, piloerection
Mental State	Alert and responsive	Mildly depressed, reduced alertness	Severely depressed, unresponsive

### Data analysis

Statistical significance was determined using GraphPad Prism version 8.0. Data are presented as the mean standard deviation (±S.D) based on a minimum of three biological replicates (n ≥ 3). For experiments with two groups, we used unpaired two-tailed Student's t-test with Welch's correction when variances were unequal. For multi-group comparisons, we employed one-way ANOVA followed by Dunnett's post-hoc test for comparisons against vehicle control. The following notation is used for *p*-values: * for *p* < 0.05, ** for *p* < 0.01, *** for *p* < 0.001, and **** for *p* < 0.0001, “ns” denotes no significant difference.

## Results

### Clinical breast milk samples effectively inhibit VACV infection.

**Figure 1.**
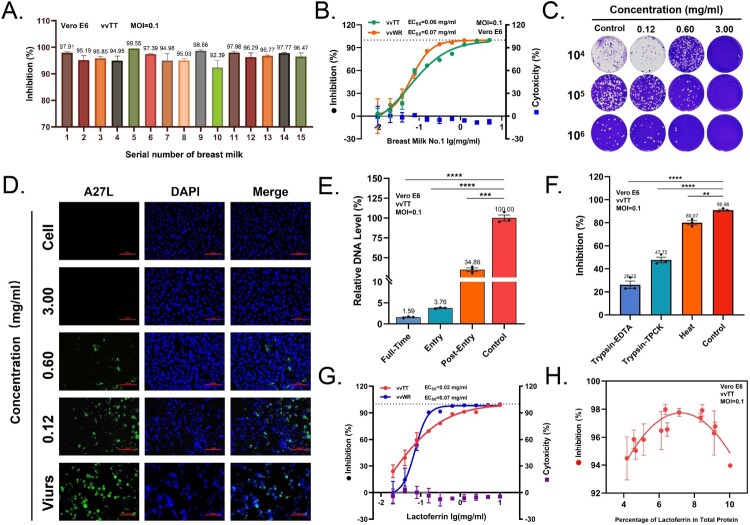
Breast milk components exhibit anti-VACV activity through heat-stable and protease-resistant mechanisms. (A) Inhibitory effects of skimmed breast milk samples (3 mg/ml total protein) from 15 healthy donors against vvTT infection (MOI = 0.1, 24 hpi) in Vero E6 cells. Viral DNA quantified by RT-qPCR and normalized to GAPDH. Data = mean ± SD (*n* = 3). (B) The dose–response curves of breast milk sample 1 against vvTT and vvWR using 10-point, 1:2 serial dilutions. EC_50_ and CC_50_ calculated using four-parameter logistic regression (GraphPad Prism 8.0). Data = mean ± SD (*n* = 3). (C) Plaque assay quantification of infectious vvTT virions after treatment with gradient breast milk concentrations (3, 0.6, 0.12 mg/ml). (D) Representative immunofluorescence images of Vero E6 cells infected with vvTT and treated with various breast milk concentrations. The DNA signal, stained with DAPI, is displayed in blue, while the visualized viral protein appears in green. Scale bars = 100 μm. (E) Time-of-addition assay with breast milk (3 mg/ml). Full-Time: present throughout infection; Entry: during 2 h adsorption only; Post-Entry: added at 2 hpi. Viral DNA quantified by RT-qPCR at 14 hpi. Data = mean ± SD (*n* = 3). ****p < 0.0001, ***p < 0.001 vs. Control (one-way ANOVA with Dunnett's test). (F) Thermolability and protease sensitivity of antiviral activity. Breast milk (0.6 mg/ml) subjected to heat treatment (100°C, 1 h) or protease digestion with trypsin-EDTA or TPCK-trypsin (250 μg/ml, 37°C, 1 h). Data = mean ± SD (*n* = 3). ****p < 0.0001, **p < 0.01 vs. Native (one-way ANOVA with Dunnett's test). (G) Dose–response curve of Lf against vvTT (MOI = 0.1, 24 hpi). Cytotoxicity of drugs shown as blue squares. The graphs depict the median with mean ± SD from a 10-point, 1:2 dilution series of lactoferrin, with *n* = 3 replicates. (H) Correlation between lactoferrin percentage of total breast milk protein and anti-vvTT activity. Lactoferrin quantified by ELISA, total protein by BCA assay. Antiviral activity measured as percent inhibition at 3 mg/ml breast milk. Linear regression with Pearson correlation coefficient shown.

To evaluate the broad-spectrum antiviral potential of breast milk, we established a vaccinia virus infection model using two representative strains: the Tiantan strain (vvTT) and the Western Reserve strain (vvWR). Breast milk samples collected from 15 healthy donors demonstrated significant inhibition against VACV infection at total protein concentrations of 3 mg/ml (Figure 1(A)). A representative sample, exhibiting EC_50_ of 0.06 mg/ml against both vvTT and vvWR (Figure 1(B)), was selected for subsequent mechanistic studies. This sample significantly reduced virus-induced cytopathic effects (CPEs) and decreased infectious viral titres from 3.1 × 10^7^ PFU/ml to 2.8 × 10^4 PFU/ml, achieving a notable 3-log10 reduction (Figure 1(C)). IFA results indicate that A27 protein production was nearly undetectable in infected cells treated with 3.0 mg/ml sample, providing compelling evidence for the potent inhibition of viral replicative capacity (Figure 1(D)). It also exhibited similar antiviral activity against vvWR, confirming efficacy across two VACV strains (Figure S1A-D). Time-of-addition assays revealed that the antiviral activity was primarily exerted during the early stages of infection, with over 95% protection when breast milk was administered 2 h prior to viral exposure (Figure 1(E)). Collectively, these results demonstrate that breast milk interferes with multiple stages of the VACV life cycle, with a pronounced effect on blocking progeny virion production.

To identify the active components in breast milk that exert antiviral effects, we systematically screened the antiviral properties of 15 breast milk-derived bioactive constituents using four cell lines representing distinct tissue tropisms (Figure S2A-B). Four protein components demonstrated greater than 90% viral inhibition at 5 mg/ml, including lactoferrin (Lf), whey protein concentrate (WPC), osteopontin (OPN), and milk fat globule membrane (MFGM). To validate the protein-dependent antiviral mechanism of breast milk, we inactivated its components through heat denaturation (100°C, 1 h), trypsin-EDTA digestion (250 μg/ml, 37°C, 1 h), and trypsin-TPCK digestion (250 μg/ml, 37°C, 1 h). All treatments significantly attenuated the inhibitory capacity of breast milk against VACV infection, with residual inhibition rates of 90.96% (±2.33%), 80.07% (±3.31%), 47.72% (±4.82%), and 26.22% (±6.40%) for control, heat-treated, trypsin-EDTA-treated and TPCK-trypsin-treated samples, respectively (Figure 1(F)). The loss of antiviral activity implicated thermolabile proteins as primary mediators of antiviral effects. We further determined the dose-dependent curves of the four proteins (Figure 1(G), Figure S2C). Among them, lactoferrin, a cationic glycoprotein secreted by exocrine glands and neutrophils [34,35], exhibited superior potency with an EC_50_ value of 0.02 mg/ml against vvTT and 0.07 mg/ml against vvWR (Figure 1(G)), outperforming the other candidates by 20- to 30- fold (Figure S2C). Furthermore, we quantified lactoferrin concentrations in various breast milk samples, ranging from 550 to 750 μg/ml, which represents 5% to 10% of the total protein content (Figure S2D), align with previously reported literature [22]. Our results revealed a biphasic relationship between the percentage of lactoferrin in total protein and antiviral potency (Figure 1(H)). Antiviral activity increased with the lactoferrin percentage up to a threshold, and then decreased, suggesting an optimal lactoferrin percentage in breast milk for maximum antiviral activity.

### Lactoferrin exhibits broad-spectrum entry blockade for orthopoxviruses

**Figure 2.**
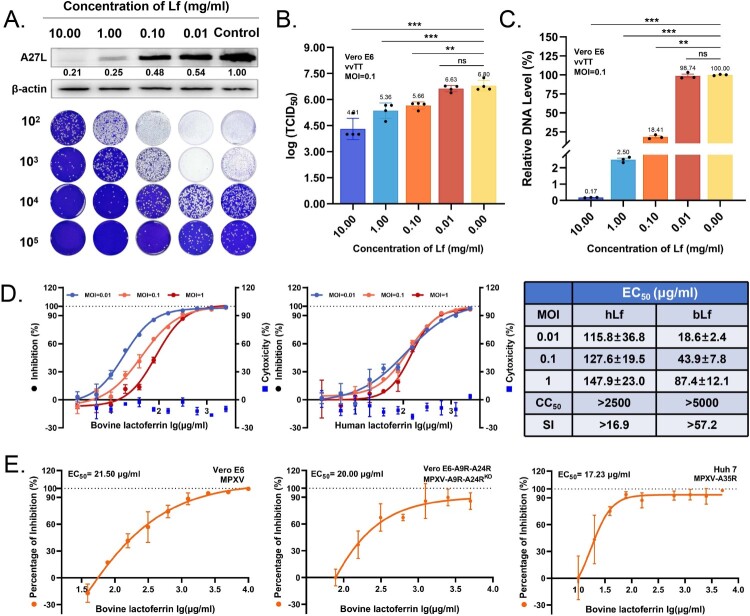
Lactoferrin demonstrates broad-spectrum anti-orthopoxvirus activity across multiple strains and infection models. (A) Western blot analysis and plaque assay quantification of vvTT replication in Vero E6 cells treated with lactoferrin (0.01-10 mg/ml). Cells were infected at MOI = 0.1 and harvested at 24 hpi. VACV A27 protein expression was detected by western blot with β-actin as loading control. Infectious viral titres were determined by plaque assay on Vero E6 monolayers. (B) Viral titres quantified by TCID_50_/ml assay following lactoferrin treatment against vvTT. TCID_50_ values were calculated using the Reed and Muench method. Data represent mean ± SD (*n* = 4). Statistical significance determined by one-way ANOVA with Dunnett's multiple comparisons test (***p < 0.001). (C) Relative viral DNA expression of vvTT quantified by RT-qPCR and normalized to GAPDH in Vero E6 cells treated with indicated lactoferrin concentrations. Data represent mean ± SD (*n* = 3). Statistical significance determined by one-way ANOVA with Dunnett's multiple comparisons test (***p < 0.001). (D) Dose–response curves of human lactoferrin and bovine lactoferrin against vvTT at MOIs of 0.01 (blue), 0.1 (orange), and 1 (red). Cytotoxicity showed as light blue scatter. (E) The dose–response curve of bovine lactoferrin against MPXV-A9R-A24R (EC_50_ = 20.00 μg/ml; 95% CI: 12.1-68.3), MPXV Clade IIb (EC_50_ = 21.50 μg/ml; 95% CI: 12.1-68.3), and MPXV-A35R pseudovirus (EC_50_ = 17.23 μg/ml; 95% CI:11.27-26.30). For authentic MPXV-trVLP and MPXV strains, viral replication was quantified by qPCR normalized to GAPDH at 48 hpi. For pseudovirus assays, entry efficiency was measured by luciferase reporter activity at 24 hpi. EC_50_ values and 95% confidence intervals were calculated using GraphPad Prism 8.0. Data represent mean ± SD (*n* = 3).

We conducted a systematic multi-modal assessment to characterize the antiviral potency of lactoferrin. The dose–response analyses demonstrated concentration-dependent inhibition of infectious particle production and viral genomic replication (Figure 2(A–C)). The administration of 10 mg/ml lactoferrin inhibited approximately 99% of virus infection. Comparative profiling of human lactoferrin (hLf) versus bovine lactoferrin (bLf) across different infection scenarios (MOI = 0.01, 0.1 and 1), the EC_50_ values of hLf were 115.8, 127.6, and 147.9 μg/ml, respectively. In comparison, bLf exhibited EC_50_ of 18.6, 43.9, and 87.4 μg/ml (Figure 2(D)). The bLf demonstrated the superior efficacy with an average EC_50_ value 2.6-fold lower than hLf, which translated to significantly higher therapeutic (SI > 57.2) compared to hLf (SI > 17.9) (Figure 2(D)). Crucially, bLf maintained potent inhibition even under high viral challenge (MOI = 1), which suggests its antiviral activity is not compromised by overwhelming viral replication pressure. Both bLf and hLf exhibited broad antiviral activity against vvTT in diverse cell types, including cervical (HeLa), liver cancer (Huh-7), and embryonic kidney (293 T) cells, with peak efficacy in HeLa cells (EC_50_ = 9.2 μg/ml) (Figure S3A-B). Cytotoxicity assays confirmed Lf maintained >90% cell viability at all tested concentrations (CC_50_ > 5000 μg) (Figure S3A-B). It positions a critical property of bLf as a promising candidate for combating rapidly progressing orthopoxvirus infections.

Extending these findings to clinically relevant pathogens, bLf exhibited robust activity against the emerging monkeypox virus (MPXV Clade IIb, IVCAS 6.9141), achieving an EC_50_ of 21.50 μg/ml (Figure 2(E)). To exclude virus-specific adaptation artefacts, we engineered a trans-complementary MPXV system based on strain MA001 from Massachusetts, USA (GenBank ON563414.3) [19], which can undergo a complete replication cycle within Vero cell lines that stably express the A9R and A24R genes (Figure S3C). The system demonstrated that bLf effectively blocked MPXV-A9R-A24R-KO replication in complementing cells with EC_50_ of 20.00 μg/ml (Figure 2(E)). Orthopoxviruses employ viral particles with distinct membrane composition and infection pathways, including intracellular mature virus particles (IMV), intracellular enveloped virus particles (IEV), and extracellular enveloped virus particles (EEV) [36–38]. We also engineered vesicular stomatitis virus (VSV) pseudotyped virus bearing key surface membrane proteins from MPXV and related orthopoxviruses to evaluate the broad-spectrum antiviral effect [39,40]. The firefly luciferase reporter gene replaced the glycoprotein gene (G) of VSV for quantitative analysis of virus infection (Figure S4A). The bLf potently inhibited MPXV-A35R pseudovirus entry into cells in a dose-dependent manner, with an EC_50_ value of 17.23 μg/ml (Figure 2(E)). In contrast, only partial suppression was observed against MPXV-M1R pseudoviruses with an EC_50_ value of 297.1 μg/ml (Figure S4B). The potency difference might reflect structural and functional variations between two membrane protein complexes. Consistent with its broad targeting capacity, bLf also neutralized VSV pseudotyped virus incorporating other MPXV membrane proteins including MPXV-B6R, MPXV-A29L, MPXV-E8L, MPXV-H3L, as well as its homologous proteins of VACV (Figure S4B). To rule out potential non-specific antiviral effects of lactoferrin's activity, we evaluated its impact on VSV replication. Lactoferrin did not inhibit VSV replication, confirming that its antiviral activity is specific to orthopoxvirus entry (Figure S4C). These findings collectively demonstrated lactoferrin's significant ability to interfere with orthopoxvirus infection across multiple particle types, highlight its broad-spectrum antiviral activity.

Considering the notable monotherapy efficacy of lactoferrin, we conducted a study to evaluate the effects of combining lactoferrin with the FDA-approved antiviral agents tecovirimat or brincidofovir. We evaluated the percentage of inhibition across an 8 × 8 matrix of dose combinations. SynergyFinder 3.0 analysis via the zero-inflated Poisson (ZIP) model [41] revealed additive interactions, with synergy scores of 1.789 (95% CI: 1.03-2.55) for lactoferrin-brincidofovir (Figure S5A-C) and 2.347 (95% CI: 1.24-3.46) for lactoferrin-tecovirimat (Figure S5D-F). Notably, subtherapeutic tecovirimat (0.2 μM) in combination with lactoferrin (125 μM) achieved equivalent efficacy to tecovirimat monotherapy (1.2 μM), demonstrating 6-fold dose reduction potential. The characteristics of the additives suggest that the immunomodulatory properties of lactoferrin can complement the effect of other antivirals without overlapping toxicity. It is a critical advantage in preventing treatment-limiting adverse effects for immunodeficient patients.

### Lactoferrin exerts an inhibitory effect on viral adsorption by binding to heparan sulphate

**Figure 3.**
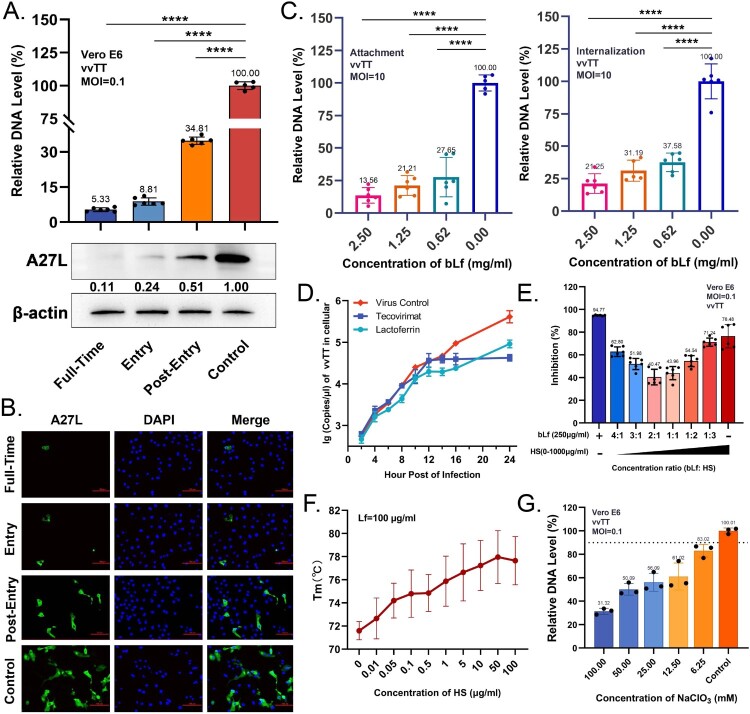
Lactoferrin inhibits orthopoxvirus infection by blocking viral entry through heparan sulphate binding. (A) Time-of-addition kinetics of 2.5 mg/ml bLf against vvTT infection (MOI = 0.1). Viral loads quantified at 14 hpi by Western blot and RT-qPCR. Data = mean ± SD (*n* = 3). ****p < 0.0001 (one-way ANOVA with Dunnett's test). (B) Representative immunofluorescence images of vvTT-infected Vero E6 cells treated with 2.5 mg/ml bLf at indicated time points. Viral A27L protein (green), nuclei (DAPI, blue). Scale bars = 100 μm. (C) The inhibitory activity of lactoferrin at concentrations of 2.5, 1.25, and 0.625 mg/ml during the entry stage was quantified using RT-qPCR. Data = mean ± SD (*n* = 3), ****p < 0.0001 (one-way ANOVA with Dunnett's test). (D) Post-entry curve depicted the replication dynamics of vvTT (MOI = 0.01) in Vero E6 cells after 2.5 mg/ml lactoferrin treatment. Tecovirimat at a concentration of 100 nM served as a positive control. Viral copies were quantified using a standard plasmid and analysed by absolute quantification methods. Data = mean ± SD (*n* = 3). (E) Heparin competition assay. Lactoferrin (250 μg/ml) pre-incubated with heparin (HS) at indicated ratios during vvTT infection (MOI = 0.1). Viral loads quantified by RT-qPCR at 24 hpi. Data = mean ± SD (*n* = 3). (F) Differential scanning fluorimetry showing direct lactoferrin-heparan sulphate binding. Thermal shift (Tm) of bLf (100 μg/ml) with increasing HS concentrations (0.02-200 μg/ml). Data = mean ± SD (*n* = 3). (G) Sodium chlorate pre-treatment depletes cell-surface HSPGs and inhibit vvTT (MOI = 0.1) infection. Viral yields quantified by RT-qPCR at 24 hpi. Data = mean ± SD (*n* = 3).

Bovine lactoferrin was used in the subsequent experiments due to its more pronounced inhibitory effect than human lactoferrin. The process of vaccinia virus entry into host cells can be divided into three distinct phases: virus attachment, hemifusion, and viral core entry [5]. The time-of-addition experiment was designed to determine the stage at which bLf inhibits vvTT infection initially. Administering 2.5 mg/ml bLf inhibits the virus entry significantly, leading to a notable reduction in virus yield by 93% (Figure 3(A,B)). Given that the virus does not internalize into cells at 4°C, a temperature shift assay was conducted to explore the specific role of lactoferrin in the viral entry mechanism [26]. It indicated that bLf inhibited vvTT during both attachment and internalization, with a more significant effect on viral adsorption (Figure 3(C)). The replication dynamics and release kinetics analysis illuminated the role of bLf following viral entry into host cells. At an MOI of 0.01, vvTT initiated replication at 4 hpi, followed by release of progeny virions at 12 hpi. Compared to tecovirimat, bLf mainly acted in the pre-assembly stage of viruses, resulting in a relatively slight reduction in viral copies in the cellular levels (Figure 3(D)). The above findings suggest that lactoferrin inhibits virus adsorption in a dose-dependent manner, with its primary mechanism of action potentially related to cell-surface attachment factors.

Attachment-blocking assays revealed that bLf pre-incubated with cells effectively inhibited the adsorption of vvTT (Figure S6A). Although the specific orthopoxvirus entry receptors remain unidentified, glycosaminoglycans (GAGs) are known to be ubiquitous cell-surface factors that facilitate viral adherence to host cells. Heparan sulphate (HS), chondroitin sulphate (CS) and dermatan sulphate (DS) facilitate initial viral adherence, while laminin acts as a bridging ligand to promote virion attachment to host cells [38,42]. To elucidate the role of attachment factors in lactoferrin-mediated antiviral activity, we supplemented cell cultures with GAGs. Lactoferrin at 250 µg/ml demonstrated approximately 90% inhibition against vvTT in the absence of HS (control group), while exogenously added HS progressively diminished the antiviral activity, reducing inhibition from 90% to approximately 40% at a 2:1 lactoferrin:heparin ratio, indicating the competitive electrostatic interactions (Figure 3(E)). Interestingly, excess HS enhanced the viral inhibition, suggesting that excess free heparin may directly interfere with viral attachment to HSPGs. We expanded our analysis to encompass additional members of the GAGs family. Similar to HS, exogenously administered CS and DS demonstrated inherent antiviral activity (Figure S6B). However, they were less effective than HS in neutralizing the antiviral activity of lactoferrin, suggesting a preferential role of HS in the lactoferrin-orthopoxvirus interaction. Differential scanning fluorimetry (DSF) confirmed direct Lf-HS interaction, with melting temperature (Tm) of Lf increasing dose-dependently with HS concentration (Figure 3(F)).

To functionally validate HSPGs as vvTT entry factors, we employed complementary enzymatic and chemical approaches. HSPGs consist of a proteoglycan core protein with covalently attached HS chains [26]. Sodium chlorate (NaClO_3_), which can reduce the sulphation of acetylated heparan [34,43], dose-dependently reduced vvTT binding (Figure 3(G)). Treatment with three heparinases cleaving HSPGs at distinct sites all reduced vvTT-cell interaction, with Heparinase I and III being the most effective (Figure S6C). Furthermore, heparinase pre-treatment (1000 mIU/ml) enhanced lactoferrin's antiviral activity, demonstrating synergistic effects (Figure S6D). Collectively, these biochemical and functional data establish HSPGs as the primary attachment factor for vvTT. It suggests that lactoferrin prevents the attachment of vvTT by competing for cell-surface HSPGs, thereby reducing subsequent viral infection of cells.

### Lactoferrin regulates the antiviral innate immune response of VACV infection

**Figure 4.**
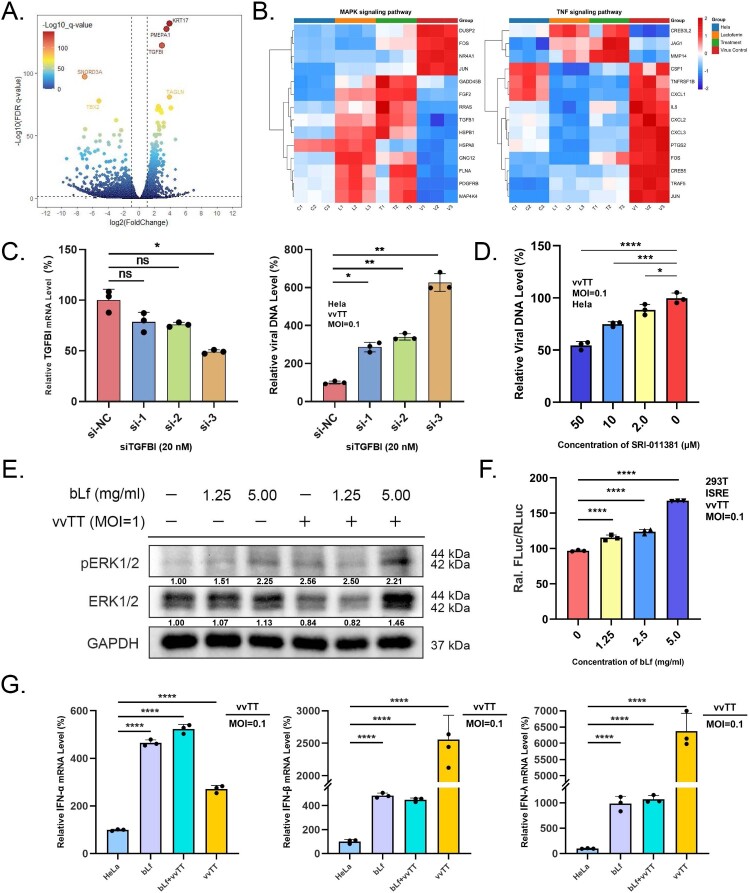
Lactoferrin enhances antiviral innate immunity through TGFBI-mediated TGF-β signalling activation. (A) Volcano plot of differentially expressed genes in vvTT-infected HeLa cells treated with 2.5 mg/ml lactoferrin compared to untreated infected cells. Downregulated genes (blue) and upregulated genes (red) with |log2(fold change)| ≥ 1 and q-value < 0.05 are highlighted. Top significantly altered genes include upregulated KRT17, PMEPA1, and TGFBI. Total: 4,131 differentially expressed genes (1,062 upregulated, 3,069 downregulated). (B) Heatmap showing expression patterns of representative genes in MAPK and TNF signalling pathways across treatment groups. Colour scale represents normalized gene expression levels (log2 transformed). (C) TGFBI knockdown enhances VACV replication. HeLa cells were transfected with TGFBI siRNA (20 nM) or scrambled control for 48 h, then infected with vvTT (MOI = 0.1). Viral loads quantified at 24 hpi by RT-qPCR normalized to GAPDH. Data = mean ± SD (*n* = 3). (D) Dose-dependent antiviral activity of SRI-011381, a TGF-β signalling agonist. Vero E6 cells were treated with SRI-011381 (2, 10, 50 μM) and infected with vvTT (MOI = 0.1). Viral loads quantified at 24 hpi by RT-qPCR normalized to GAPDH. Data = mean ± SD (*n* = 3). (E) Western blot analysis of ERK and pERK expression in HeLa cells under indicated conditions. GAPDH served as loading control. Band intensities quantified using ImageJ. Representative blot and quantification from three independent experiments shown. Data = mean ± SD (*n* = 3). (F) Dose-dependent activation of interferon-stimulated response element (ISRE) promoter activity by lactoferrin. 293 T cells were transfected with ISRE-luciferase reporter, infected with vvTT (MOI = 0.1), and treated with lactoferrin (1.25, 2.5, 5.0 mg/ml) for 24 h. Luciferase activity measured by dual-luciferase assay and normalized to Renilla control. Data = mean ± SD (*n* = 3). (G) RT-qPCR quantification of type I (IFN-α, IFN-β) and type III (IFN-λ) interferon mRNA expression in HeLa cells treated with lactoferrin (2.5 mg/ml, 24 h) with or without vvTT infection (MOI = 0.1). Expression normalized to GAPDH. Data = mean ± SD (*n* = 3).

To investigate the molecular mechanisms underlying lactoferrin's antiviral activity, we performed RNA-seq on HeLa cells following VACV infection with or without bLf treatment (Figure S7A). The qPCR analysis confirmed efficient viral infection and lactoferrin-mediated antiviral effects (Figure S7B). Comparative transcriptomic analysis revealed that lactoferrin treatment induced substantial gene expression changes relative to vvTT-infected controls, with 4,131 differentially expressed genes (DEGs) identified (1,062 upregulated, 3,069 downregulated; |log2FC| ≥ 1, q < 0.05) (Figure 4(A), Figure S7C). The key transcription factors (p53, c-Fos, c-Jun, EGR1) validation confirmed that lactoferrin not only mildly induced their expression but normalized VACV-induced hyperactivation to baseline levels (Figure S7D-E). Comparative analysis with published MPXV infection datasets revealed conserved modulation of these orthopoxvirus-host transcriptional interfaces [44,45]. Pathway enrichment analysis using KEGG and Gene Ontology databases demonstrated significant regulation of innate immune pathways, particularly the MAPK and TNF signalling cascades (Figure 4(B)), alongside enrichment of genes involved in focal adhesion, actin cytoskeleton regulation, and neutrophil extracellular trap formation (Figure S7F). Notably, VACV infection-induced robust upregulation of pro-inflammatory mediators, including chemokines (CXCL2, CXCL3, CXCL16), cytokines (IL-6, IL-11), and growth factors (EREG, AREG), which are known to facilitate viral replication [46,47]. Lactoferrin treatment effectively reversed these infection-induced transcriptional changes and additionally upregulated BGN, GSN, TAGLN, and TGFBI (Figure S8A-C).

Among the identified genes, TGFBI emerged as a particularly virus-responsive target, whose expression was suppressed during VACV infection but significantly restored upon lactoferrin treatment (Figure S8C). Since TGFBI is a downstream effector of TGF-β signalling, we hypothesized that the observed TGFBI upregulation may reflect lactoferrin-mediated activation of TGF-β-dependent antiviral responses. Function experiments found that siRNA-mediated TGFBI knockdown significantly enhanced VACV replication (Figure 4(C)), demonstrating that TGFBI is essential for cellular antiviral responses. Correspondingly, treatment with SRI-011381, a TGF-β signalling agonist, dose-dependently inhibited vvTT infection (Figure 4(D)), providing pharmacological support for the protective role of this pathway. These complementary experiments genetically validate the importance of TGFBI in host defense against orthopoxviruses.

Given the link between TGF-β signalling and antiviral pathways (MAPK/ERK, JAK/STAT, and NF-κB cascade), we investigated the underlying mechanism [48]. Western blot analysis revealed that VACV infection suppressed expression of ERK and STAT3 as an immune evasion strategy. Lactoferrin treatment effectively restored these protein levels and enhanced their phosphorylation (Figure 4(E), Figure S9A). To determine whether MAPK activation contributes to entry inhibition, we explore the antiviral stage of anisomycin, a well-established MAPK pathway activator. While anisomycin treatment post-viral entry significantly reduced viral replication, it showed no inhibitory effect when added during the viral entry phase (Figure S9B). This result indicates that lactoferrin's potent entry-blocking activity is likely mediated through MAPK-independent mechanisms, while MAPK signalling may contribute to antiviral effects at post-entry stages. To further characterize the antiviral mechanisms of lactoferrin, we performed qPCR analysis and dual-luciferase reporter assays. Lactoferrin dose-dependently induced expression of both type I (IFN-α, IFN-β) and type III (IFN-λ) interferons, and activated the interferon-stimulated response element (ISRE) promoter consistent with enhanced antiviral signalling (Figure 4(F,G)). We analysed interferon-stimulated gene (ISG) expression using the Molecular Signatures Database (MSigDB) gene sets. It revealed that lactoferrin treatment upregulated multiple ISGs, including CFH, TENT5A, IFITM1, TRAFD1, CCL2, and PLA2G4A, compared to cell controls (Figure S9C). Hierarchical clustering demonstrated that Lf treatment established a distinct ISG expression signature separate from infected conditions. Collectively, these findings suggest that lactoferrin enhances antiviral defense through the TGFBI-TGF-β signalling axis. Lactoferrin treatment restores TGFBI expression and activates downstream MAPK/ERK and JAK2/STAT3 cascades, leading to interferon and ISG production. This signalling cascade contributes to counteracting VACV-induced immunosuppression and establishing an antiviral state in host cells.

### Lactoferrin exhibits therapeutic efficacy against VACV infection in vivo

**Figure 5.**
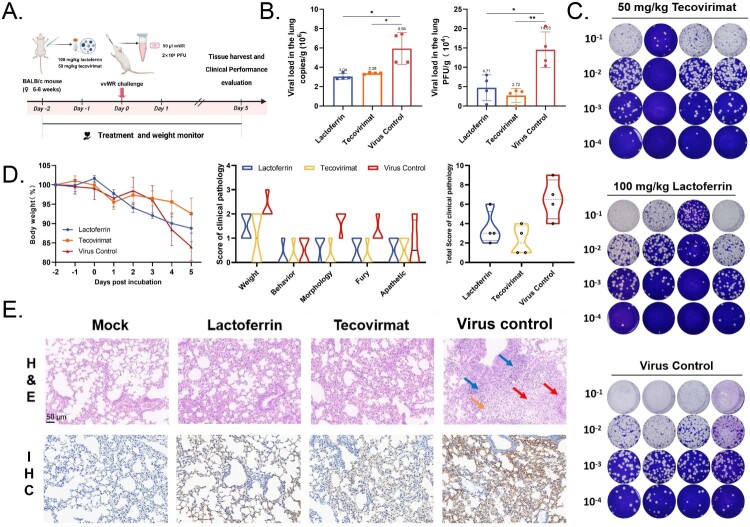
Bovine lactoferrin exhibits therapeutic efficacy against VACV infection *in vivo*. (A) Experimental design for prophylactic bLf treatment. (B) Quantification of viral load in lung tissues. Viral genome copies were quantified as genomes per gram of tissue (left), while virus titres were calculated as PFU per gram of tissue (right). The data analysis performed using GraphPad Prism version 8.0. Data = mean ± SD (*n* = 4), *p < 0.05, **p < 0.01 (two-tailed *t*-test). (C) Quantitative analysis of infectious viral particles in the lung with 100 mg/kg lactoferrin, 50 mg/kg tecovirimat and PBS treatment based on plaque assay. (D) Weight loss dynamics and survival rates monitored daily for 7 days and clinical and pathological scoring of mice. (E) H&E staining and IHC analysis of the lung sections. The sections exhibited diffuse degeneration and necrosis of the epithelial lining (black arrows), along with haemorrhage, edema (red arrows), and fibrin exudation into the surrounding alveoli (blue arrows). IHC analysis conducted using a human anti-VACV A27L monoclonal antibody (brown signal). Mock was the blank control group without virus infection. The slides were digitized using a Pannoramic MIDI histoscanner (3DHISTECH), and the images analysed with CaseViewer software. Scale bars, each representing 50 micrometers, are provided for reference in each image.

The therapeutic effects of bLf were evaluated on VACV infection mouse model (Figure 5(A)). The virus control group exhibited notable weight loss and clinical signs, including lethargy, reduced activity, decreased food consumption, poor fur quality, and increased sensitivity to cold. Analyses performed on day 5 post-infection showed that bLf and tecovirimat treatment reduced viral genomic in the lung tissues by ∼3 × 10^6^ copies /g and decreased infectious viral titres from 1.45 × 10^5^ to 4.71 × 10^4^ PFU/ g, as determined by RT-qPCR and plaque assay, respectively (Figure 5(B,C)). Furthermore, bLf administration modestly relieved weight loss (16% reduction in infectious group vs. 10% reduction in the Lf-treated group), and reduced the average clinical pathological scores decreasing from 6.50 to 3.50 (Figure 5(D)). H&E staining revealed that lung tissues from the virus-infected group displayed extensive necrotic areas, with loss of bronchiolar and alveolar structures, appearing as amorphous eosinophilic material. Necrotic cell debris was evident, along with interstitial edema around blood vessels, loosely arranged connective tissue, and significant infiltration of granulocytes, lymphocytes, and macrophages. Macrophages were also observed within the bronchiolar lumen. The eosinophil counts and immune cells infiltration in the lungs of mice treated with lactoferrin or tecovirimat diminished, highlighting the protective effect of lactoferrin on pulmonary health (Figure 5(E)). Moreover, lactoferrin demonstrated protective efficacy by reducing infectious viral particle burden in lung tissues, confirming entry blockade by lactoferrin against orthopoxviruses *in vivo*. Immunohistochemical analysis of A27L viral protein showed significant reduction in viral antigen-positive cells in both tecovirimat- and lactoferrin-treated groups compared to PBS controls (Figure 5(E)), consistent with the presence of residual viral genomes and infectious particles observed by qPCR and plaque assay (Figure 5(E)). These findings confirmed the inhibitory effect and therapeutic potential of lactoferrin against vvWR in mice, suggesting that it can be used as a clinical intervention.

## Discussion

Our systematic evaluation identified lactoferrin as the most potent anti-orthopoxvirus component in breast milk. As a widely available dietary supplement with established safety, lactoferrin exhibits multi-modal inhibition across diverse infection models [34]. The correlation between lactoferrin concentration and breast milk antiviral potency suggests potential applications in lactoferrin-fortified infant formulas. We provide the first demonstration of lactoferrin's dual anti-orthopoxvirus mechanisms: blocking HSPG-dependent viral entry and activating host innate immunity via TGFBI-TGF-β signalling.

Lactoferrin competitively antagonizes orthopoxvirus attachment by binding to HSPGs, the primary viral adhesion receptors that interact with positively charged viral envelope proteins through electrostatic interactions [38,42]. Multiple lines of evidence support this mechanism: exogenous HS reverses lactoferrin's antiviral efficacy in a dose-dependent manner, while enzymatic HS removal or sulphation inhibition enhances viral blocking by over 70%. DSF analysis confirms direct high-affinity lactoferrin-HS binding. Notably, bLf exhibits superior efficacy compared to hLf, likely due to higher cationic charge in the N-lobe region (pI 8.7 vs. 8.0), which strengthens electrostatic interactions with HSPGs and viral glycoproteins. This species-specific difference has important implications for therapeutic development and supplementation strategies. While these findings establish lactoferrin as an HSPG-targeted entry inhibitor, future studies using HSPG-deficient cell lines are needed to distinguish direct viral neutralization from competitive HS occupation mechanisms.

Beyond lactoferrin's broad-spectrum antiviral activity against HIV, HBV, HSV, and SARS-CoV-2 through conserved mechanisms such as blocking HSPG-mediated virion binding and receptor competition [23,25,26], we identified an orthopoxvirus-specific immunomodulatory pathway involving TGFBI-mediated restoration of interferon responses. RNA-seq analysis of VACV-infected cells revealed that lactoferrin selectively modulates host responses by suppressing pro-inflammatory cascades while enhancing interferon-mediated defenses. Lactoferrin downregulated inflammatory chemokines (CXCL2, CXCL3, CXCL16) and suppressed AREG/EGFR signalling [49–52], protecting against cytokine storms without compromising antiviral immunity [49,51,53–59]. Critically, lactoferrin counteracted VACV-induced immunosuppression by upregulating TGFBI, which activated TGF-β-dependent MAPK/ERK and JAK2/STAT3 cascades [48,52]. Western blot confirmed restoration of VACV-suppressed ERK and STAT3 phosphorylation. Functionally, TGFBI knockdown enhanced viral replication, while TGF-β agonist SRI-011381 recapitulated lactoferrin's antiviral effects, demonstrating the sufficiency of this pathway for antiviral defense.

Time-of-addition experiments using anisomycin revealed that MAPK activation inhibits viral replication only post-entry, indicating that lactoferrin's entry-blocking activity is MAPK-independent and instead mediated through direct HSPG competition. MAPK signalling likely contributes to post-entry antiviral effects. RNA-seq identified upregulation of entry-restricting ISGs (IFITM1) and immune modulators (CCL2, CSF1), establishing lactoferrin as a multi-stage antiviral agent. These findings position lactoferrin as a host-directed immunotherapeutic that reverses virus-induced immunosuppression through the TGF-β-interferon axis. This mechanism may circumvent viral resistance and provides rationale for combination therapy with tecovirimat to simultaneously target viral replication and restore immune function.

Lactoferrin is available as a 250 mg supplement and designated “Generally Recognized as Safe” (GRAS) by the FDA [34]. However, a critical consideration for clinical translation is whether the observed *in vitro* effective concentrations (EC_50_ = 12.5-50 µg/ml) are physiologically attainable. Systemic administration via intraperitoneal or intravenous routes achieves therapeutic plasma concentrations [60], with lactoferrin distributing to macrophage-rich organs including lungs via LRP1/LFR receptors [61]. Our murine VACV model demonstrated that systemic lactoferrin reduced pulmonary viral loads and infectious titres, prevented necrotizing bronchopneumonia, and eliminated viral antigen-positive cells with efficacy comparable to tecovirimat. However, short plasma half-lives (12.6 min IV, 7 h IP) necessitate frequent dosing or formulation optimization [62,63]. Future studies should measure lactoferrin concentrations in bronchoalveolar lavage fluid and lung tissue to validate therapeutic exposure. Oral administration faces bioavailability challenges, with absolute bioavailability of ∼1% due to gastrointestinal degradation [62]. Animal studies achieve serum concentrations of 0.036-2.1 µg/mL at high doses (200-2000mg/kg) [64], potentially insufficient for direct antiviral activity but adequate for mucosal defense and TGFBI-mediated immunomodulation. Recombinant human lactoferrin demonstrates superior bioavailability with peak serum levels at 3–6 h and immune activation evidenced by elevated IL-18 [65]. Notably, serum lactoferrin increases from 0.4–2.0 µg/mL at baseline to 200 µg/mL during infections [66], suggesting prophylactic potential at mucosal sites. Clinical evidence supports lactoferrin's therapeutic utility. Combined with interferon, lactoferrin manages high viral loads in chronic hepatitis C [67]. COVID-19 patients receiving liposomal bovine lactoferrin experienced faster symptom resolution than standard care [35]. Multiple studies demonstrate lactoferrin's ability to modulate immune responses and suppress pro-inflammatory cytokines including IL-6 [68].

Despite advancements in antiviral development, novel therapeutics require years to reach clinical application [45,69]. Current orthopoxvirus treatments face limitations of that few smallpox drugs effectively inhibit MPXV, and DNA polymerase inhibitors carry renal or bone marrow toxicity [12,13]. Human breast milk offers an attractive source of naturally occurring antivirals with established safety profiles, enabling rapid clinical translation. Epidemiological data reveal critical gaps in current therapeutic approaches. Approximately 52% of MPXV cases occur in HIV-infected individuals, with 25% experiencing advanced immunosuppression (https://www.who.int/), correlating with increased hospitalization and mortality. MPXV pathogenesis in these populations involves impaired NK-cell function, lymphopenia, cytokine storms, and potential antibody-dependent enhancement [70]. Consequently, our findings in immunocompetent models may not fully represent clinical outcomes in immunocompromised patients, particularly HIV-positive individuals where co-infection exacerbates disease severity [70]. Future studies should evaluate lactoferrin efficacy in T-cell-deficient or chronically inflamed animal models, assessing effects on viral replication, lesion formation, and vaccine responses. Validation with emerging Clade I MPXV strains will be essential to establish broad-spectrum activity across variants of public health concern. Additionally, clinical translation must consider both virus-induced immune dysfunction and drug-related organ toxicity to optimize therapeutic strategies for vulnerable populations [71].

Combination therapy represents a promising strategy for MPXV treatment [72,73]. We demonstrate that lactoferrin produces additive antiviral effects when combined with brincidofovir or tecovirimat through complementary mechanisms. Lactoferrin blocks viral entry via heparan sulphate binding and restores host immunity through TGF-β pathways, while tecovirimat or brincidofovir inhibit viral egress and DNA replication, respectively. This mechanistic orthogonality may enable dose reduction of direct-acting antivirals while maintaining efficacy, potentially mitigating toxicity and addressing resistance in immunocompromised patients. However, our combination data are limited to single-dose ratios evaluated *in vitro*. The relationship between *in vitro* additive effects and clinical dose-sparing depends on pharmacokinetics, tissue distribution, and host immune status, which differ substantially between cell culture and patients. Future studies should systematically evaluate dose–response matrices across multiple combination regimens and validate these findings in murine challenge models to assess whether additive effects translate to enhanced survival, accelerated viral clearance, and reduced pathology *in vivo*.

Another limitation of our study is the absence of direct tissue lactoferrin quantification, leaving uncertainty about whether *in vitro* effective doses are attainable in target organs. To address oral bioavailability constraints, future formulation strategies should explore intranasal delivery, receptor-mediated nanocarriers, or sustained-release formulations. Local mucosal delivery represents the most promising approach for orthopoxvirus infections transmitted via respiratory droplets and contact. Intranasal administration directly targets upper respiratory infection sites with high mucosal concentrations, while nebulized delivery optimizes airway surface exposure to prevent pulmonary complications. These routes provide both direct virucidal effects via HSPG blockade and local immune enhancement, offering advantages over systemic administration and warranting prioritization in clinical development.

Lactoferrin's safety profile as a naturally occurring milk protein positions it favourably for rapid clinical development against emerging orthopoxvirus threats. Our findings demonstrate a multi-targeted mechanism offering significant advantages over conventional antivirals regarding resistance barriers. By targeting host heparan sulphate receptors rather than viral proteins, lactoferrin reduces escape mutation likelihood, as viruses cannot readily alter conserved host–pathogen interactions without compromising fitness. Additionally, lactoferrin modulates host innate immunity through the TGF-β-interferon axis, creating dual selective pressure. This host-directed approach is inherently less prone to resistance than viral enzyme inhibitors, as viruses must simultaneously overcome both HSPG-mediated entry blockade and immune restoration. The observed additive effects with tecovirimat suggest combination therapy could further reduce resistance emergence by targeting orthogonal mechanisms. This positions lactoferrin as a resistance-resilient strategy, particularly valuable given limited orthopoxvirus treatment options and emerging tecovirimat resistance [68]. Future studies should optimize lactoferrin formulations to enhance bioavailability and extend therapeutic duration for clinical translation.

## Supplementary Material

SI Appendix Figure.pdf

Supporting Information R2.docx
