# Supplementary material for: Lactoferrin blocks orthopoxvirus entry via heparan sulphate and regulates host antiviral pathways
Source: Emerg Microbes Infect. 2026 Mar 6;15(1):2631205. doi: 10.1080/22221751.2026.2631205 (PMC12973843; doi:10.1080/22221751.2026.2631205)
Supplement: SI Appendix Figure.pdf [file TEMI_A_2631205_SM3975.pdf]

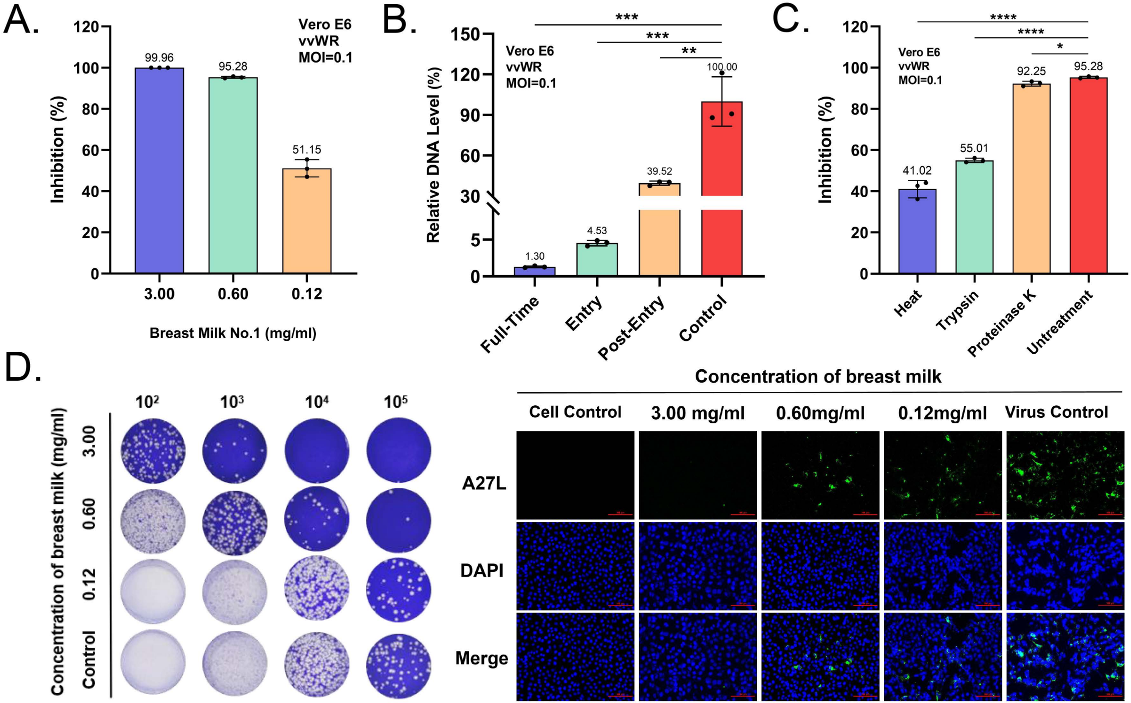
A.
Vero E6
vvWR
MOI=0.1
Inhibition (%)
99.96
95.28
51.15
3.00
0.60
0.12
Breast Milk No.1 (mg/ml)
B.
Vero E6
vvWR
MOI=0.1
Relative DNA Level (%)
***
***
**
100.00
39.52
4.53
1.30
Full-Time
Entry
Post-Entry
Control
C.
Vero E6
vvWR
MOI=0.1
Inhibition (%)
****
****
*
41.02
55.01
92.25
95.28
Heat
Trypsin
Proteinase K
Untreatment
D.
Concentration of breast milk (mg/ml)
10^2
10^3
10^4
10^5
3.00
0.60
0.12
Control
Concentration of breast milk
Cell Control
3.00 mg/ml
0.60mg/ml
0.12mg/ml
Virus Control
A27L
DAPI
Merge

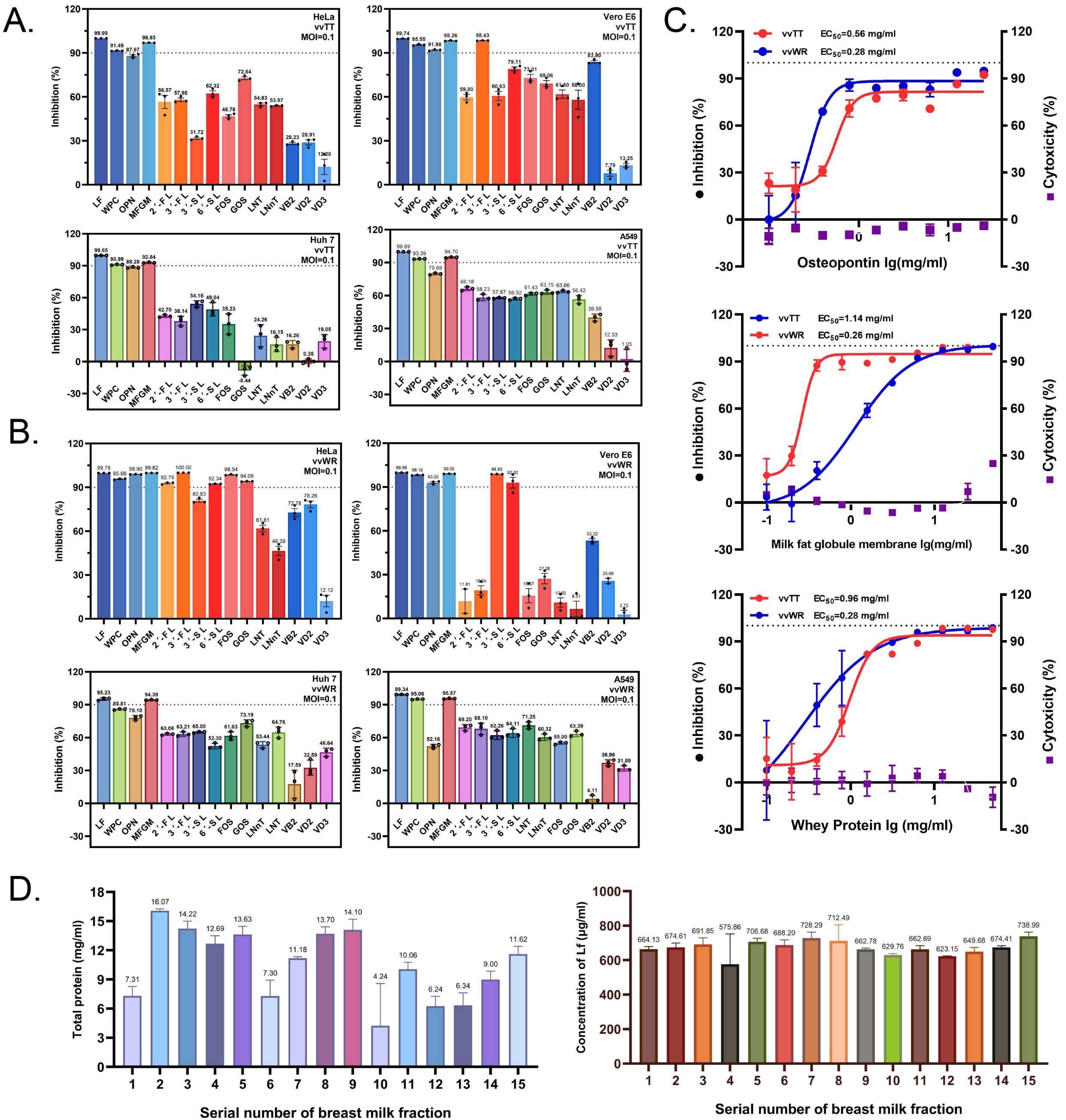
A.
HeLa
vvTT
MOI=0.1
Vero E6
vvTT
MOI=0.1
Huh 7
vvTT
MOI=0.1
A549
vvTT
MOI=0.1
Inhibition (%)
LF WPC OPN MFGM 2'-FL 3'-FL 3'-SL 6'-SL FOS GOS LNT LNnT VB2 VD2 VD3
B.
HeLa
vvWR
MOI=0.1
Vero E6
vvWR
MOI=0.1
Huh 7
vvWR
MOI=0.1
A549
vvWR
MOI=0.1
C.
vvTT EC50=0.56 mg/ml
vvWR EC50=0.28 mg/ml
Osteopontin lg(mg/ml)
vvTT EC50=1.14 mg/ml
vvWR EC50=0.26 mg/ml
Milk fat globule membrane lg(mg/ml)
vvTT EC50=0.96 mg/ml
vvWR EC50=0.28 mg/ml
Whey Protein lg (mg/ml)
● Inhibition (%)
Cytoxicity (%)
D.
Total protein (mg/ml)
Serial number of breast milk fraction
Concentration of Lf (μg/ml)
Serial number of breast milk fraction

A.

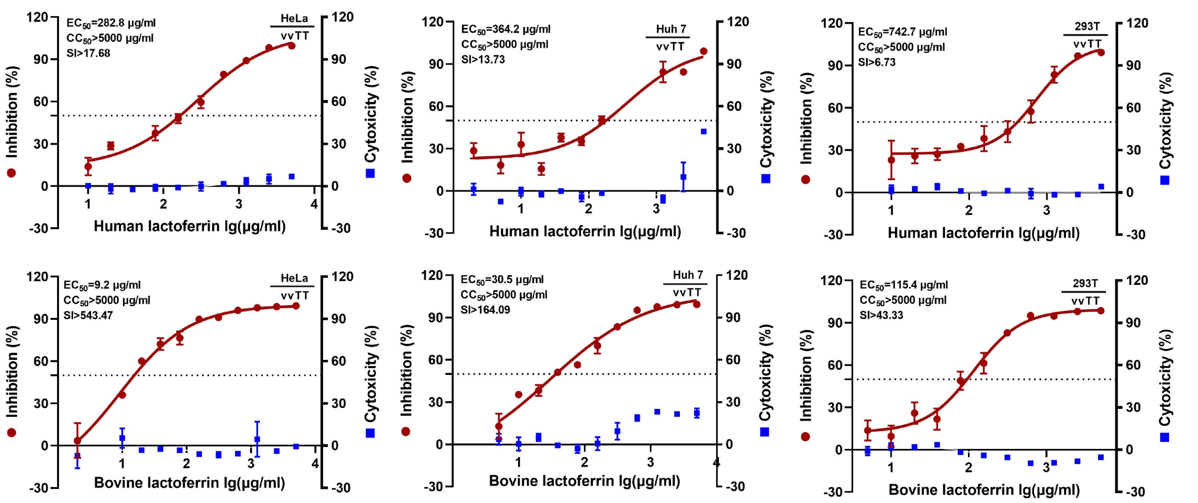

B.

Cell		hLf	bLf
CC_{50}(μg/ml)		>5000	>5000
HeLa	EC_{50}(μg/ml)	282.8±75.0	9.2±6.3
	SI	>17.68	>543.48
Huh 7	EC_{50}(μg/ml)	364.2±200.8	30.5±25.0
	SI	>13.73	>148.37
293T	EC_{50}(μg/ml)	742.7±137.0	115.4±21.0
	SI	>6.75	>43.3

C.

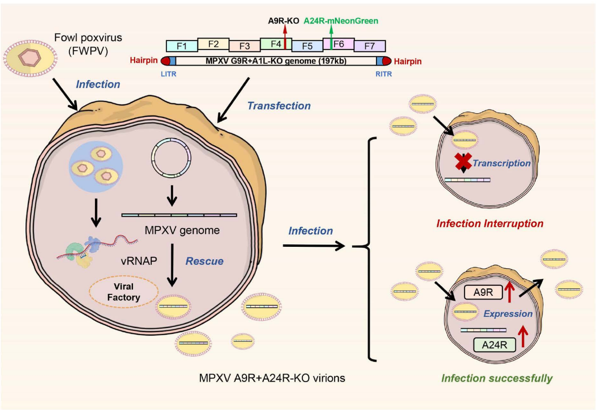

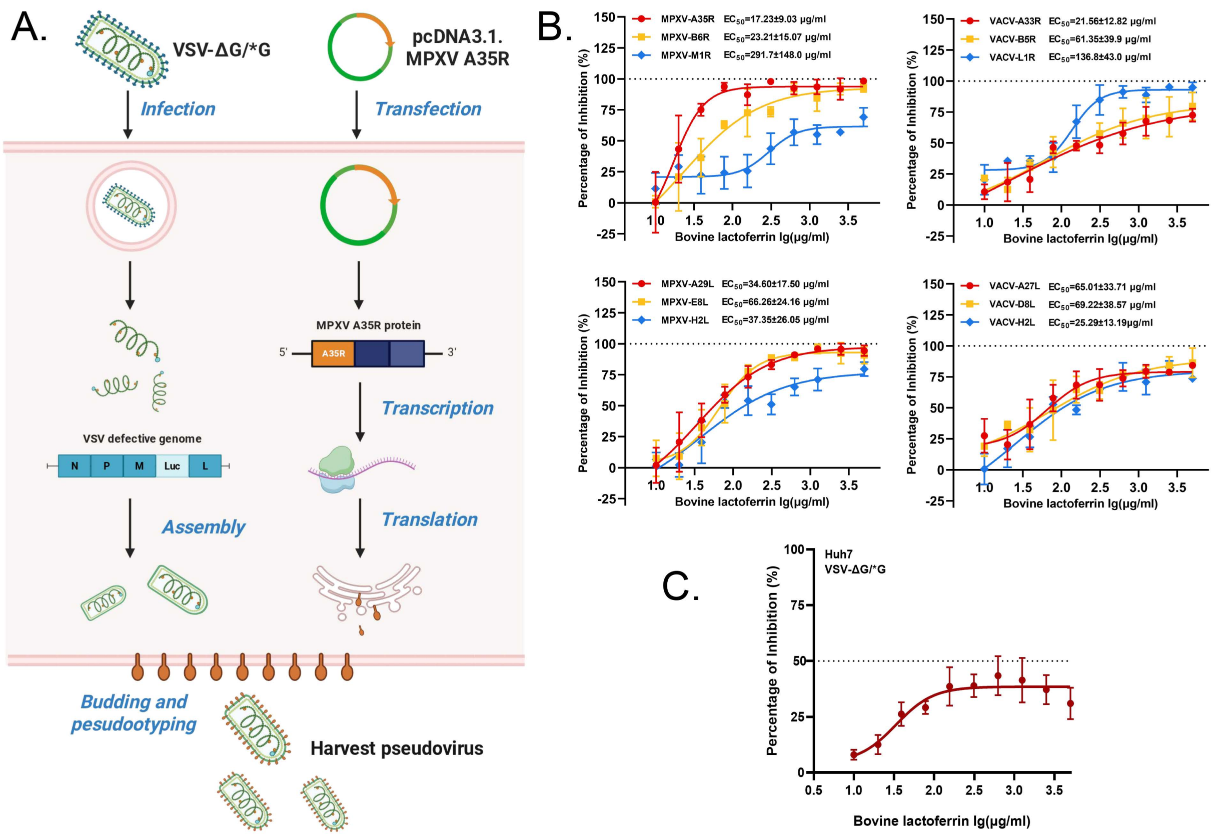

A.
VSV-ΔG/*G
pcDNA3.1. MPXV A35R
Infection
Transfection
MPXV A35R protein
5'
A35R
3'
Transcription
VSV defective genome
N
P
M
Luc
L
Assembly
Translation
Budding and pesudootyping
Harvest pseudovirus
B.
MPXV-A35R EC50=17.23±9.03 μg/ml
MPXV-B6R EC50=23.21±15.07 μg/ml
MPXV-M1R EC50=291.7±148.0 μg/ml
VACV-A33R EC50=21.56±12.82 μg/ml
VACV-B5R EC50=61.35±39.9 μg/ml
VACV-L1R EC50=136.8±43.0 μg/ml
MPXV-A29L EC50=34.60±17.50 μg/ml
MPXV-E8L EC50=66.26±24.16 μg/ml
MPXV-H2L EC50=37.35±26.05 μg/ml
VACV-A27L EC50=65.01±33.71 μg/ml
VACV-D8L EC50=69.22±38.57 μg/ml
VACV-H2L EC50=25.29±13.19μg/ml
Percentage of Inhibition (%)
Bovine lactoferrin lg(μg/ml)
C.
Huh7
VSV-ΔG/*G
Percentage of Inhibition (%)
Bovine lactoferrin lg(μg/ml)

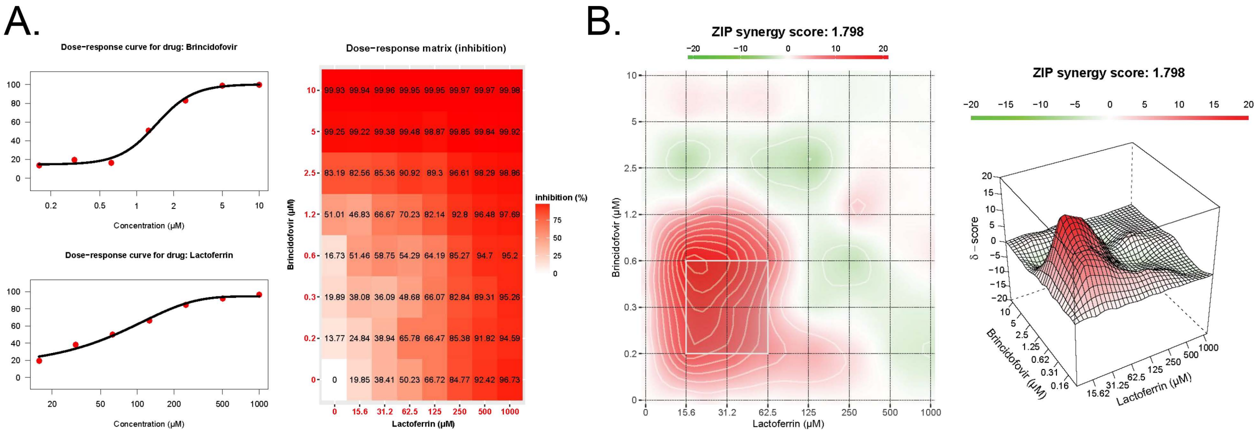

C.

Drug combination	Synergy score	Most synergistic area score	Method	95% CI	Weight
Lactoferrin-Brincidofovir	1.798±0.76	10.853	ZIP	1.23	1.384

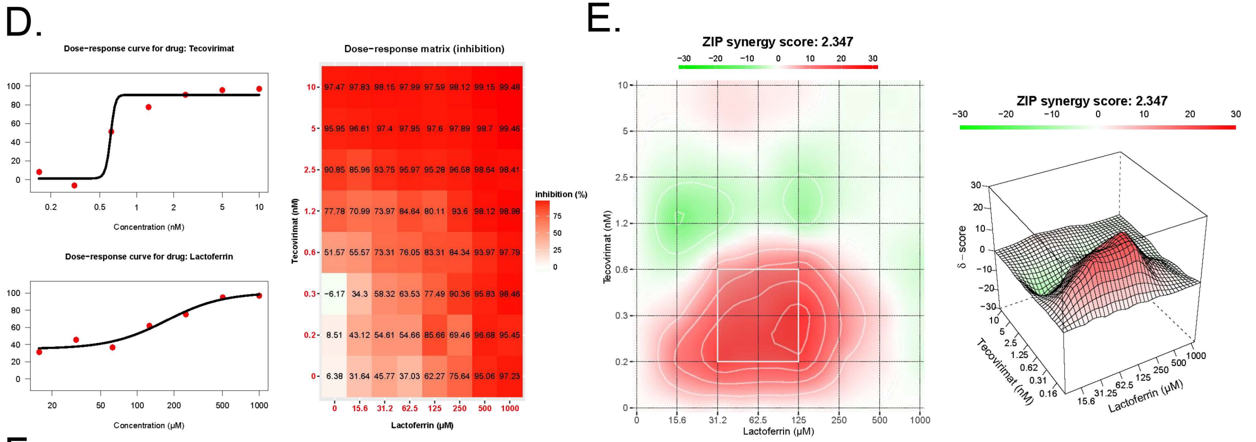

F.

Drug combination	Synergy score	Most synergistic area score	Method	95% CI	Weight
Lactoferrin-Tecovirimat	2.347±1.11	15.369	ZIP	1.11	1.384

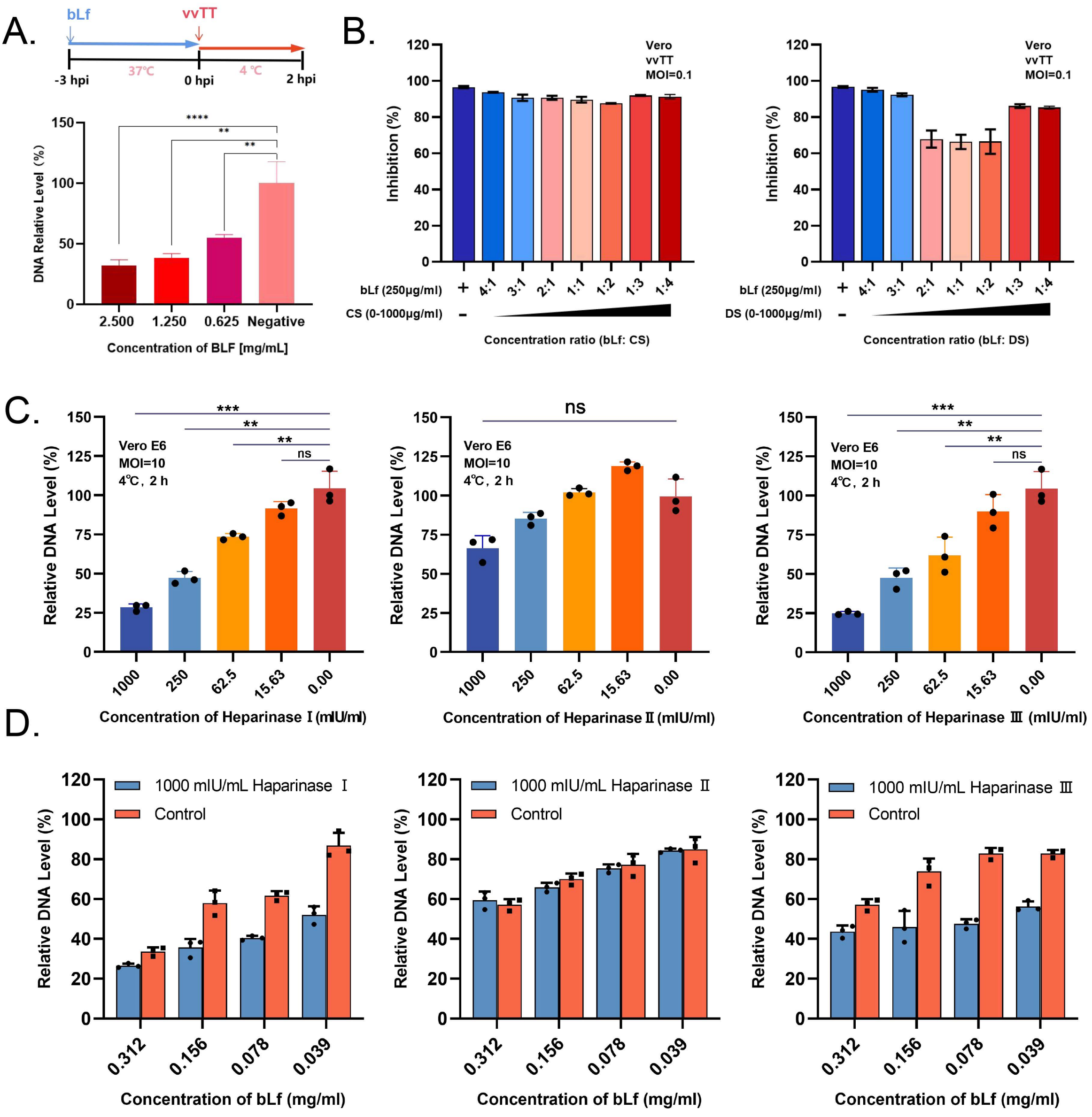

A.
bLf
vvTT
37℃
4 ℃
-3 hpi
0 hpi
2 hpi
DNA Relative Level (%)
Concentration of BLF [mg/mL]
2.500
1.250
0.625
Negative
****
**
**
B.
Vero
vvTT
MOI=0.1
Inhibition (%)
bLf (250μg/ml)
CS (0-1000μg/ml)
DS (0-1000μg/ml)
Concentration ratio (bLf: CS)
Concentration ratio (bLf: DS)
4:1
3:1
2:1
1:1
1:2
1:3
1:4
C.
Vero E6
MOI=10
4℃, 2 h
Relative DNA Level (%)
Concentration of Heparinase Ⅰ (mIU/ml)
Concentration of Heparinase Ⅱ (mIU/ml)
Concentration of Heparinase Ⅲ (mIU/ml)
1000
250
62.5
15.63
0.00
***
**
**
ns
D.
1000 mIU/mL Haparinase Ⅰ
1000 mIU/mL Haparinase Ⅱ
1000 mIU/mL Haparinase Ⅲ
Control
Relative DNA Level (%)
Concentration of bLf (mg/ml)
0.312
0.156
0.078
0.039

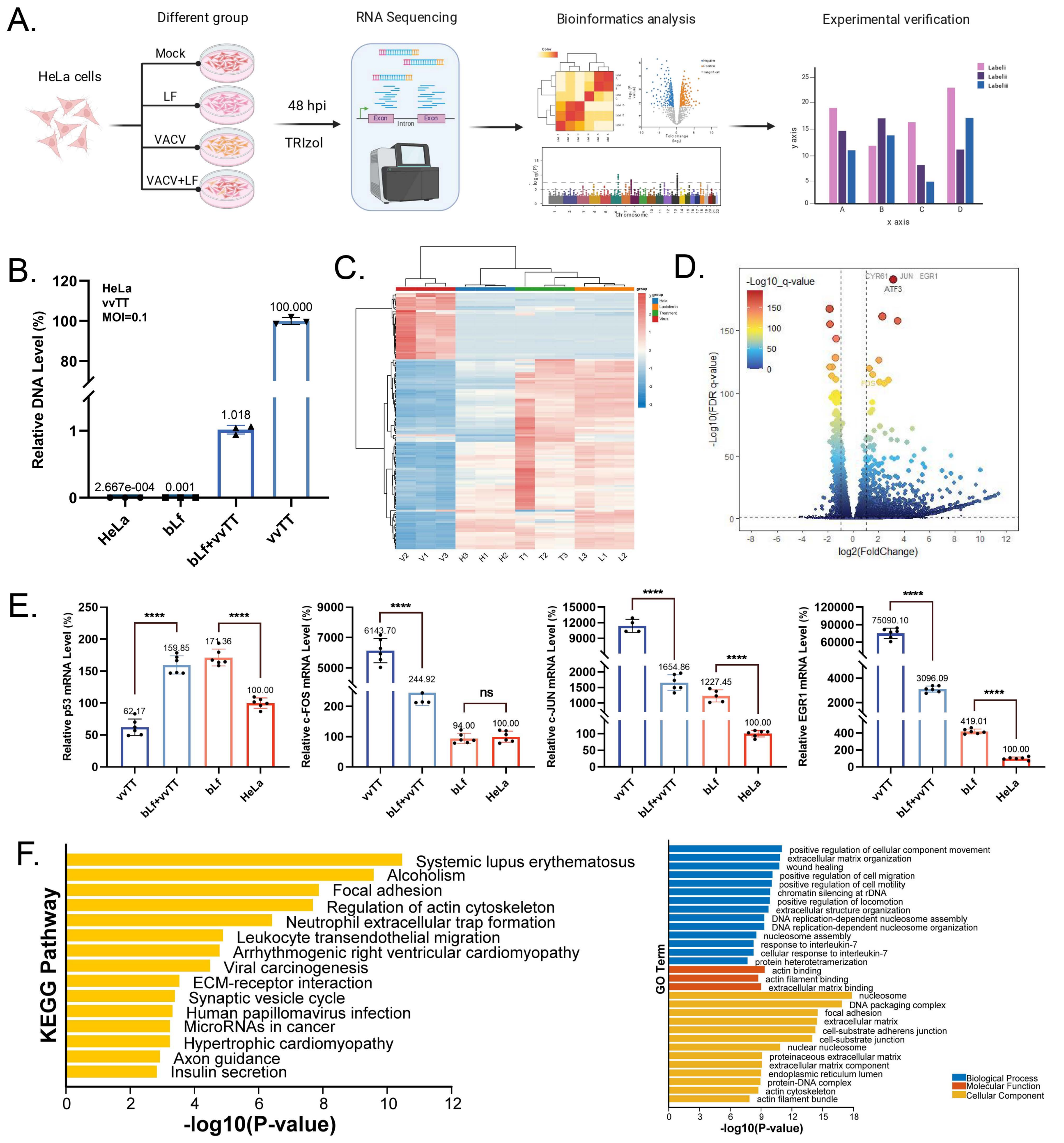

A.
Different group
RNA Sequencing
Bioinformatics analysis
Experimental verification
HeLa cells
Mock
LF
VACV
VACV+LF
48 hpi
TRIzol
B.
HeLa
vvTT
MOI=0.1
Relative DNA Level (%)
2.667e-004
0.001
1.018
100.000
HeLa
bLf
bLf+vvTT
vvTT
C.
D.
-Log10_q-value
CYR61
JUN
EGR1
ATF3
FOS
-Log10(FDR q-value)
log2(FoldChange)
E.
Relative p53 mRNA Level (%)
Relative c-FOS mRNA Level (%)
Relative c-JUN mRNA Level (%)
Relative EGR1 mRNA Level (%)
62.17
159.85
171.36
100.00
6143.70
244.92
94.00
100.00
ns
1654.86
1227.45
75090.10
3096.09
419.01
F.
KEGG Pathway
Systemic lupus erythematosus
Alcoholism
Focal adhesion
Regulation of actin cytoskeleton
Neutrophil extracellular trap formation
Leukocyte transendothelial migration
Arrhythmogenic right ventricular cardiomyopathy
Viral carcinogenesis
ECM-receptor interaction
Synaptic vesicle cycle
Human papillomavirus infection
MicroRNAs in cancer
Hypertrophic cardiomyopathy
Axon guidance
Insulin secretion
-log10(P-value)
GO Term
positive regulation of cellular component movement
extracellular matrix organization
wound healing
positive regulation of cell migration
positive regulation of cell motility
chromatin silencing at rDNA
positive regulation of locomotion
extracellular structure organization
DNA replication-dependent nucleosome assembly
DNA replication-dependent nucleosome organization
nucleosome assembly
response to interleukin-7
cellular response to interleukin-7
protein heterotetramerization
actin binding
actin filament binding
extracellular matrix binding
nucleosome
DNA packaging complex
focal adhesion
extracellular matrix
cell-substrate adherens junction
cell-substrate junction
nuclear nucleosome
proteinaceous extracellular matrix
extracellular matrix component
endoplasmic reticulum lumen
protein-DNA complex
actin cytoskeleton
actin filament bundle
Biological Process
Molecular Function
Cellular Component

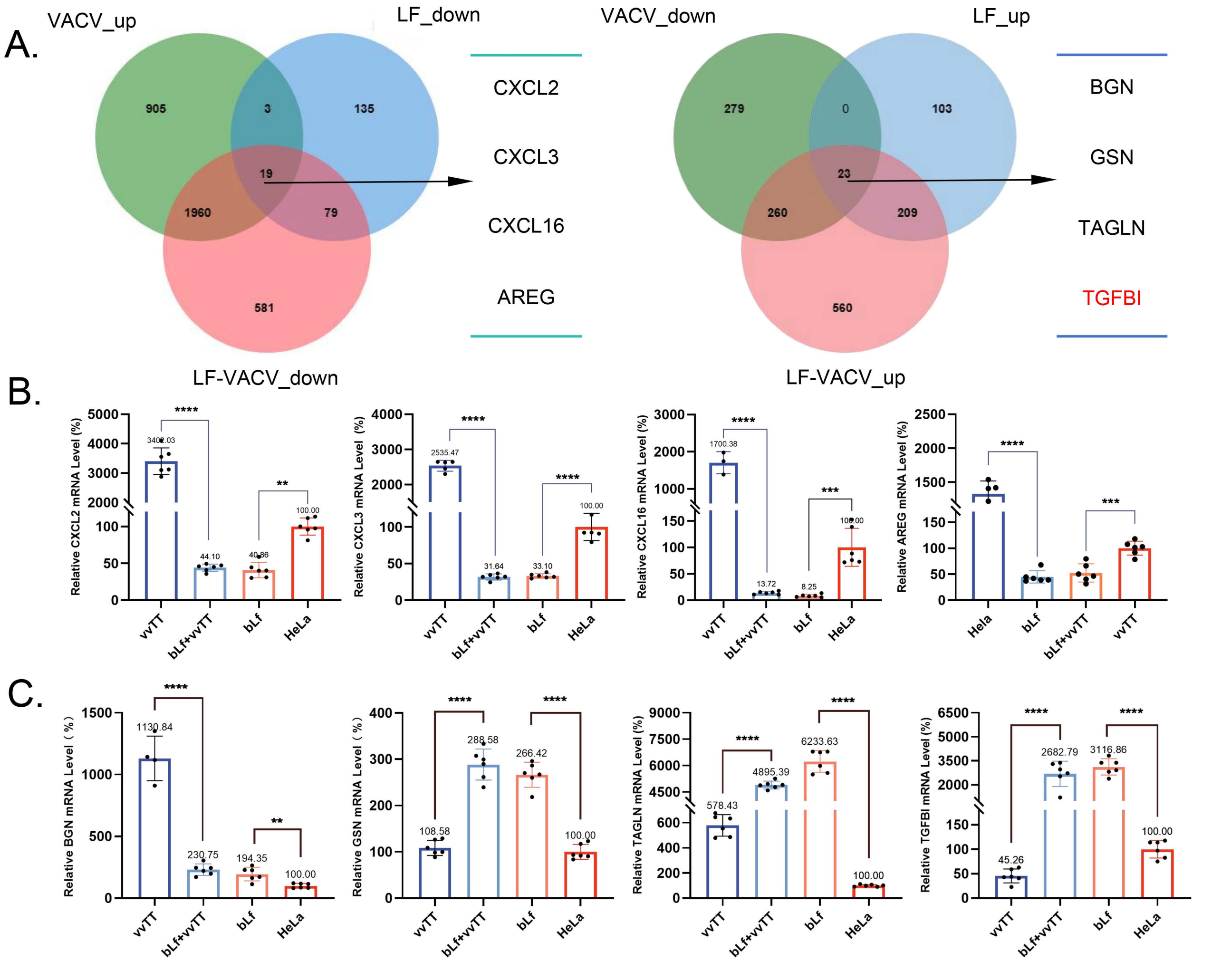

A.
VACV_up
LF_down
905
3
135
19
1960
79
581
LF-VACV_down
CXCL2
CXCL3
CXCL16
AREG
VACV_down
LF_up
279
0
103
23
260
209
560
LF-VACV_up
BGN
GSN
TAGLN
TGFBI
B.
Relative CXCL2 mRNA Level (%)
Relative CXCL3 mRNA Level (%)
Relative CXCL16 mRNA Level (%)
Relative AREG mRNA Level (%)
vvTT
bLf+vvTT
bLf
HeLa
Hela
C.
Relative BGN mRNA Level (%)
Relative GSN mRNA Level (%)
Relative TAGLN mRNA Level (%)
Relative TGFBI mRNA Level (%)
1130.84
230.75
194.35
100.00
108.58
288.58
266.42
578.43
4895.39
6233.63
45.26
2682.79
3116.86

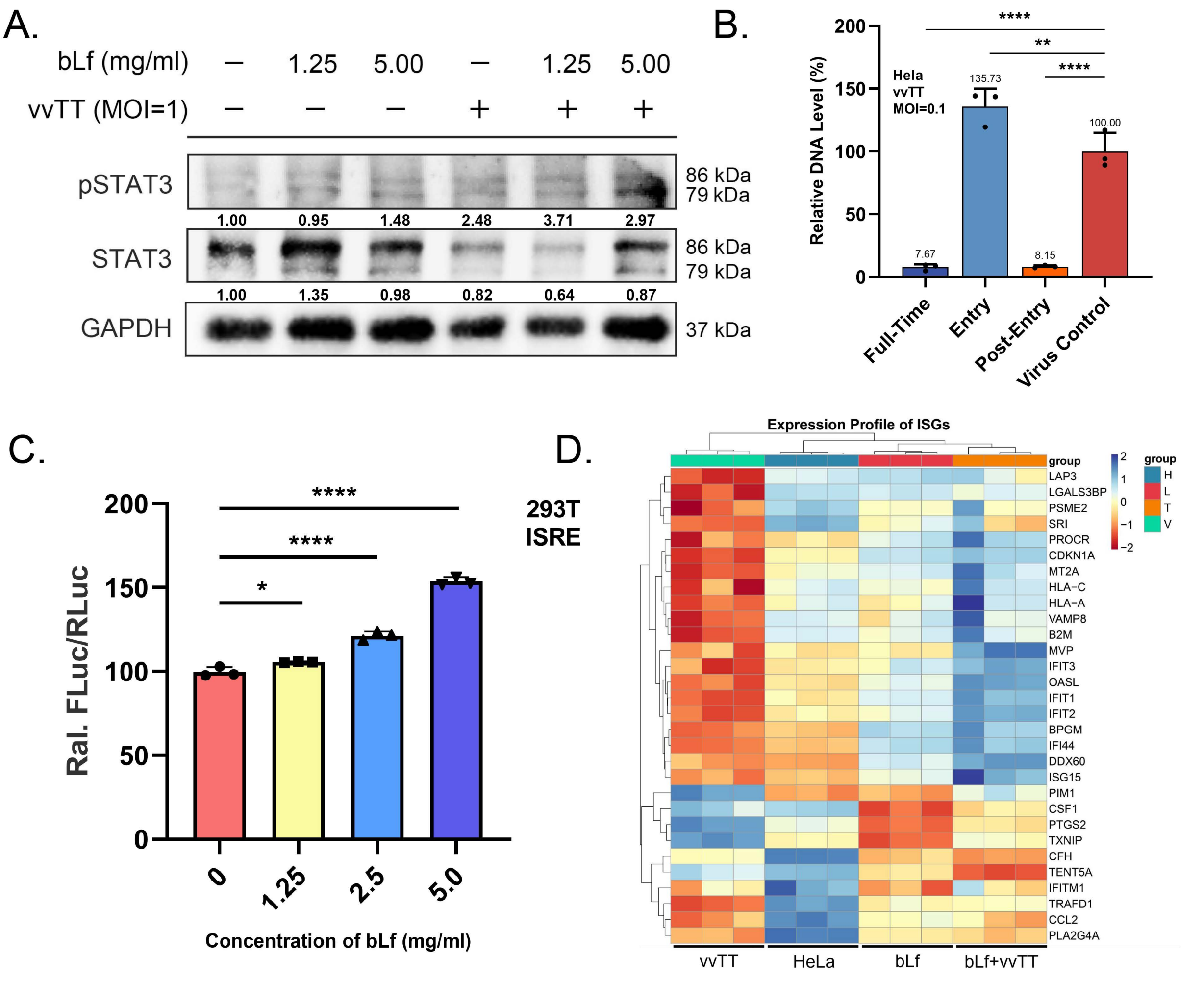
A.
bLf (mg/ml) – 1.25 5.00 – 1.25 5.00
vvTT (MOI=1) – – – + + +
pSTAT3
86 kDa
79 kDa
1.00 0.95 1.48 2.48 3.71 2.97
STAT3
86 kDa
79 kDa
1.00 1.35 0.98 0.82 0.64 0.87
GAPDH
37 kDa
B.
Hela
vvTT
MOI=0.1
Relative DNA Level (%)
****
**
****
7.67
135.73
8.15
100.00
Full-Time
Entry
Post-Entry
Virus Control
C.
293T
ISRE
Ral. FLuc/RLuc
****
****
*
Concentration of bLf (mg/ml)
D.
Expression Profile of ISGs
group
LAP3
LGALS3BP
PSME2
SRI
PROCR
CDKN1A
MT2A
HLA−C
HLA−A
VAMP8
B2M
MVP
IFIT3
OASL
IFIT1
IFIT2
BPGM
IFI44
DDX60
ISG15
PIM1
CSF1
PTGS2
TXNIP
CFH
TENT5A
IFITM1
TRAFD1
CCL2
PLA2G4A
group
H
L
T
V
vvTT
HeLa
bLf
bLf+vvTT
