# Supplementary material for: Lactoferrin blocks orthopoxvirus entry via heparan sulphate and regulates host antiviral pathways
Source: Emerg Microbes Infect. 2026 Mar 6;15(1):2631205. doi: 10.1080/22221751.2026.2631205 (PMC12973843; doi:10.1080/22221751.2026.2631205)
Supplement: Supporting Information R2.docx [file TEMI_A_2631205_SM3974.docx]

Supporting Information


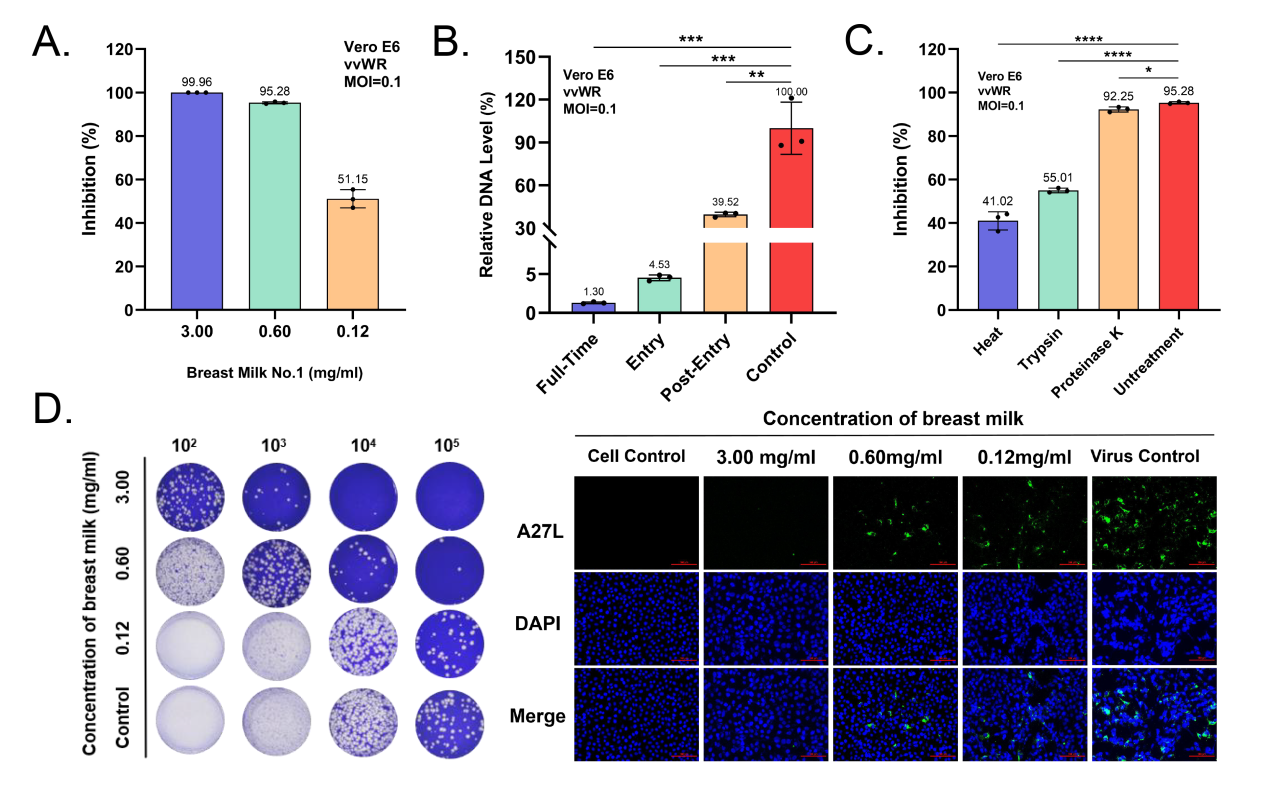


**Figure S1. Breast milk and lactoferrin exhibit consistent antiviral activity against vaccinia virus Western Reserve strain.** (A) Dose-dependent inhibitory effects of skimmed breast milk (3, 0.6, 0.12 mg/ml total protein) against vvWR infection (MOI=0.1, 24 hpi) in Vero E6 cells. Viral DNA quantified by RT-qPCR normalized to GAPDH. Data = mean ± SD (n=3). (B) Time-of-addition assay with 3 mg/ml breast milk against vvWR infection (MOI=0.1). Full-Time: present throughout infection; Entry: during 2 h adsorption only; Post-Entry: added at 2 hpi. Viral DNA quantified by RT-qPCR at 14 hpi. Data = mean ± SD (n=3). ***p<0.001, **p<0.01 vs. Virus control (one-way ANOVA with Dunnett's test). (C) Residual antiviral activity of breast milk (0.6 mg/ml) against vvWR after heat treatment (100°C, 1 h) or protease digestion with trypsin-EDTA or Proteinase K (250 μg/ml, 37°C, 1 h). Data = mean ± SD (n=3). ****p<0.0001, *p<0.05 vs. Native ( one-way ANOVA with Dunnett's test). (D) Plaque assay quantification and representative immunofluorescence images of vvWR-infected Vero E6 cells treated with gradient breast milk concentrations (3, 0.6, 0.12 mg/ml). Viral protein (green), nuclei (DAPI, blue). Scale bars = 100 μm.


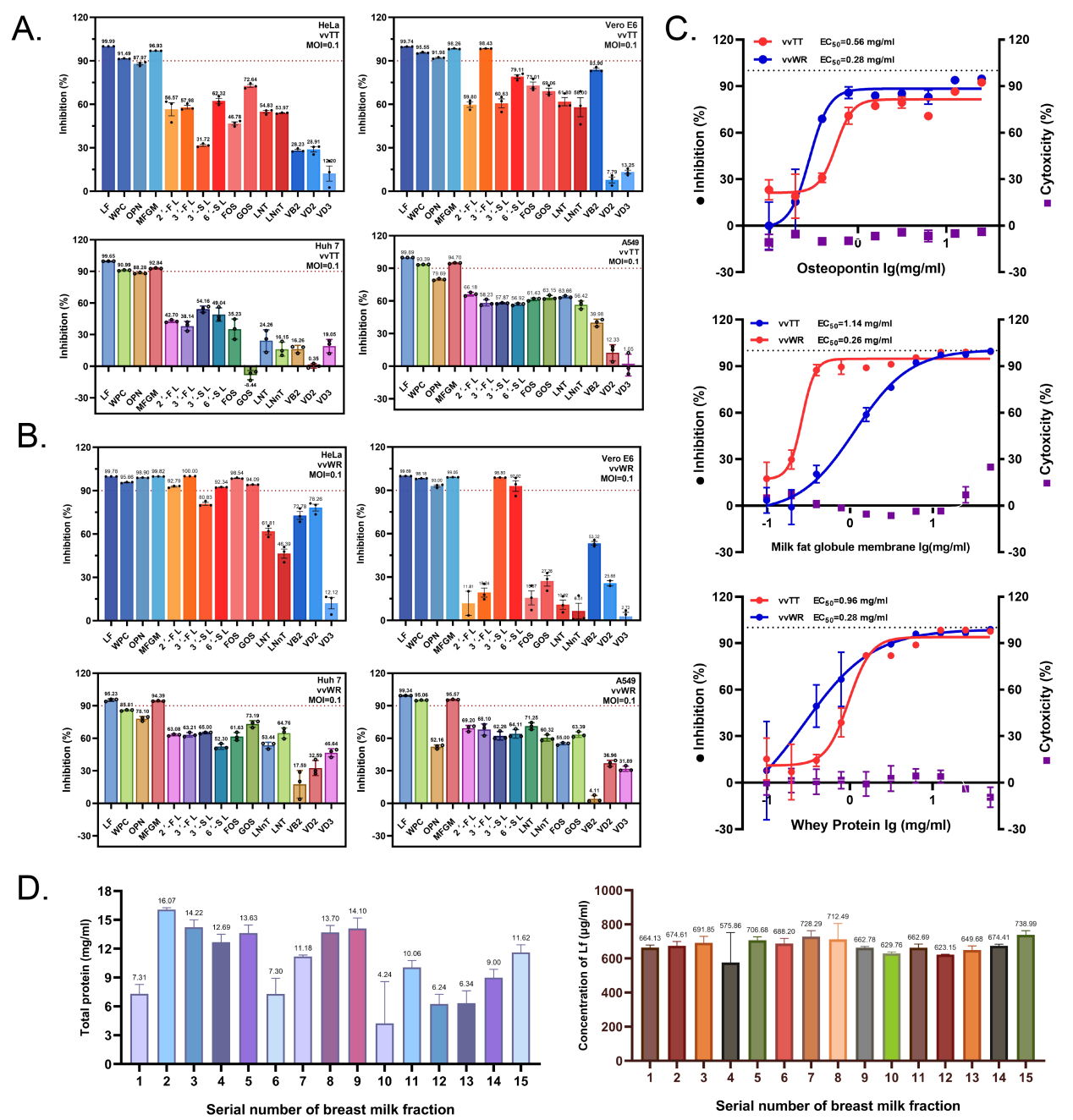


**Figure S2. Systematic screening identifies lactoferrin as the most potent antiviral component in breast milk.** (A) Antiviral activity of 15 breast milk-derived bioactive constituents against vvTT (MOI=0.1, 24 hpi) across four cell lines (HeLa, Vero E6, Huh7, A549). Components tested at 5 mg/ml included 4 proteins (LF, WPC, OPN, MFGM), 8 human milk oligosaccharides (2'-FL, 3'-FL, 3'-SL, 6'-SL, FOS, GOS, LNT, LNnT), and 3 vitamins (VB2, VD2, VD3). Viral DNA quantified by RT-qPCR normalized to GAPDH. Data = mean ± SD (n=3). (B) Antiviral activity of the same 15 breast milk components against vvWR (MOI=0.1, 24 hpi) across four cell lines. Experimental conditions identical to panel A. Data = mean ± SD (n=3). (C) Dose-response curves of OPN, MFGM, and WPC against vvTT (red curves) and vvWR (blue curves) using 10-point, 1:2 serial dilutions. Cytotoxicity shown as purple squares. EC_50_ and CC_50_ calculated using four-parameter logistic regression (GraphPad Prism 8.0). Data = mean ± SEM (n=3). (D) Quantification of lactoferrin concentration and total protein content in breast milk samples from 15 donors. Lactoferrin measured by ELISA, total protein by BCA assay. Lactoferrin represents 30-50% of total protein content (3-4 mg/ml). Data = mean ± SD (n=4).


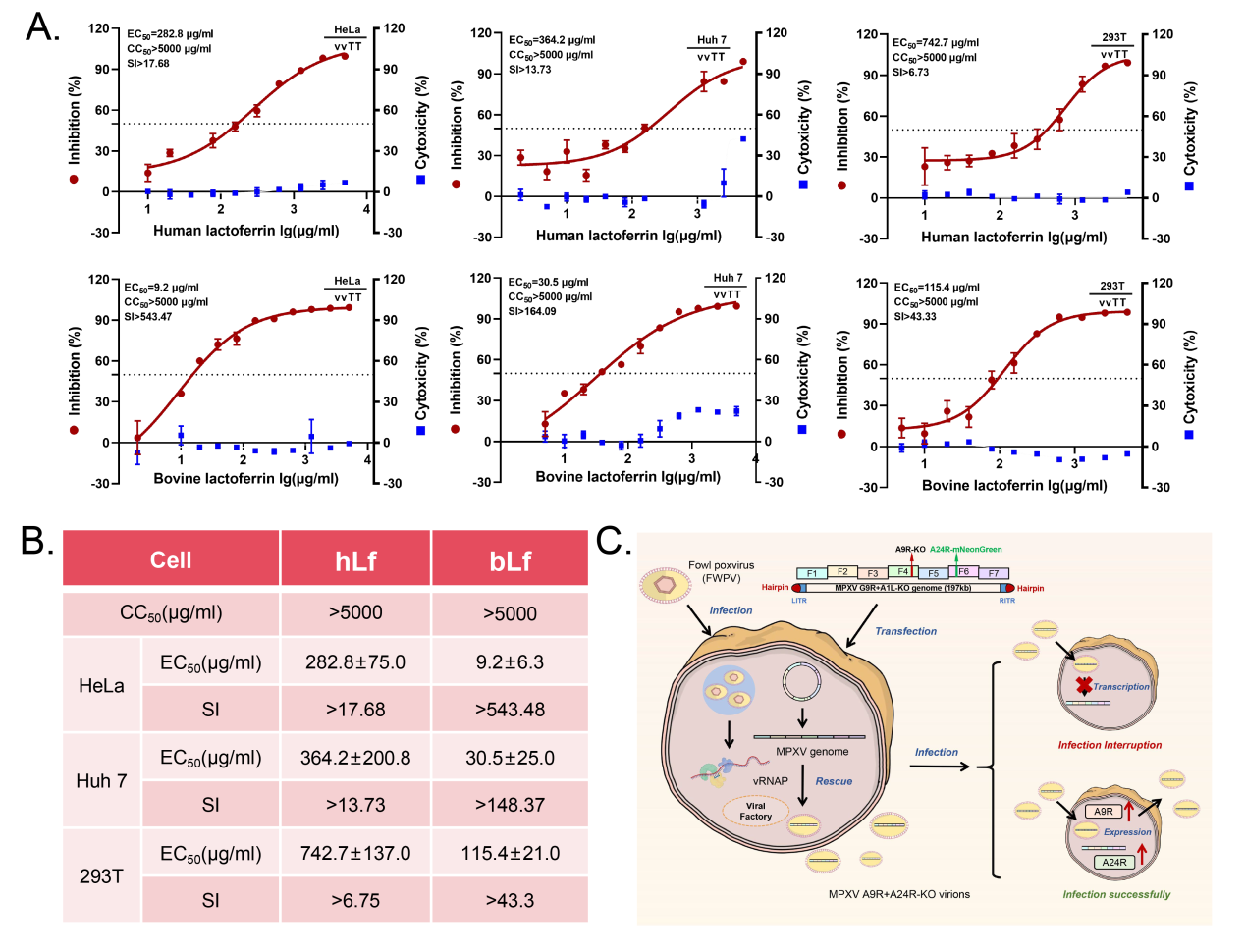


**Figure S3. Lactoferrin demonstrates broad-spectrum antiviral activity across diverse cell types and against engineered monkeypox virus.** (A) Dose-response curve of hLf and bLf against vvTT (MOI = 0.01, 36 hpi) in HeLa, Huh 7, and 293T cells using 10-point, 1:2 serial dilution. Viral DNA quantified by RT-qPCR normalized to GAPDH (dark red curves). Cell viability measured by CellTiter-Blue assay (blue squares). EC_50_ and CC_50_ calculated using four-parameter logistic regression (GraphPad Prism 8.0). Data = mean ± SD (n=3). (B) Summary table of antiviral potency, cytotoxicity, and selectivity indices for hLf and bLf against vvTT across three cell lines. EC_50_, CC_50_, and selectivity index (SI = CC50/EC50) values presented for HeLa, Huh7, and 293T cells. (C) Schematic illustration of trans-complementary MPXV system construction. Full-length MPXV MA001 genome (GenBank: ON563414.3) assembled from seven synthetic fragments with precise deletion of A9R and A24R genes and insertion of fluorescent protein reporters. Engineered MPXV-A9R-A24R-KO strain completes replication cycle exclusively in Vero cells stably expressing A9R and A24R proteins, enabling viral entry and morphogenesis studies.

**
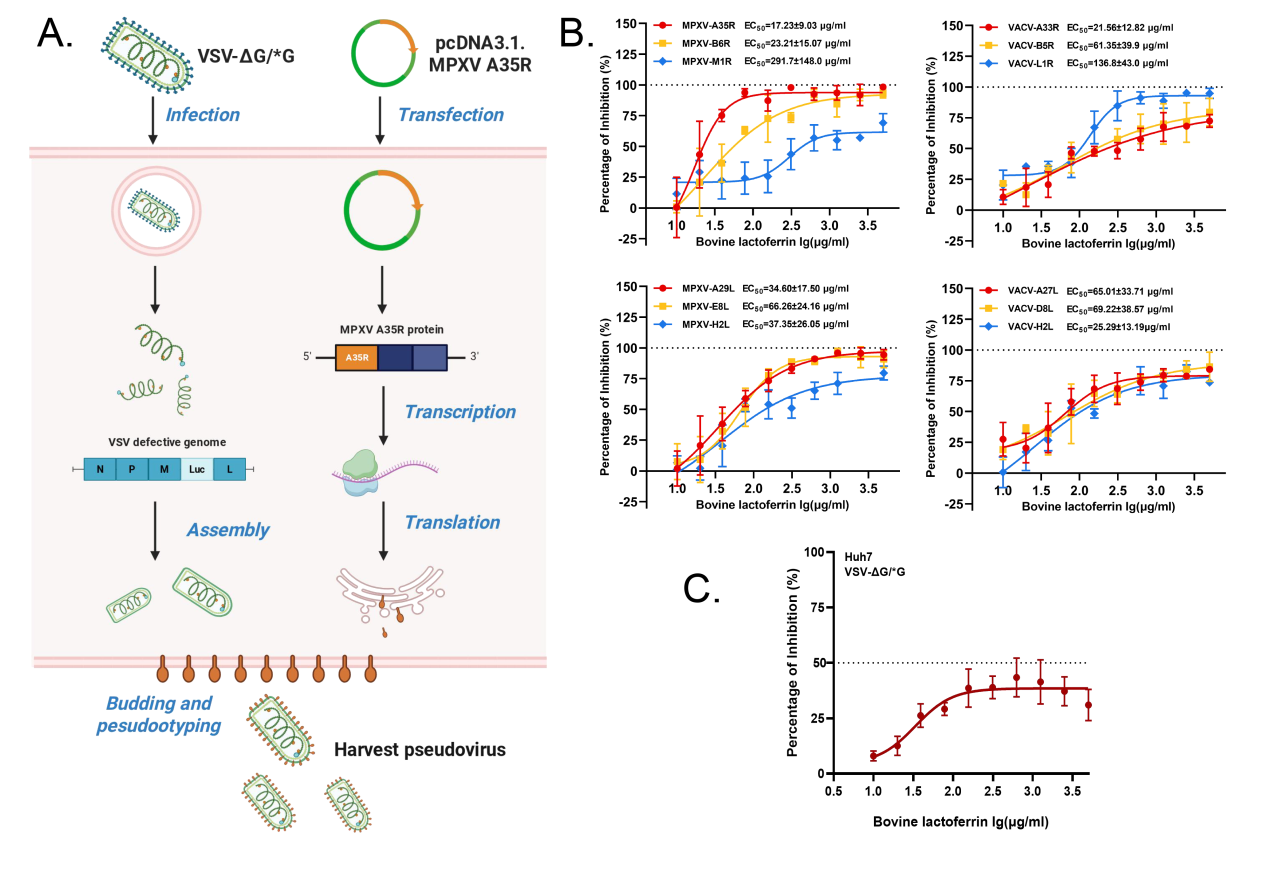
**

**Figure S4. Lactoferrin inhibits pseudovirus particles with orthopoxvirus membrane protein infection but not VSV genome replication in Huh-7 cells.** (A) Schematic of the VSV-based orthopoxvirus pseudovirus production system. 293T cells were transfected with plasmids encoding individual MPXV/VACV membrane proteins for envelope display, then infected with VSV G*ΔG in which the VSV G gene is deleted and replaced by a firefly luciferase reporter for quantitative infection readout. Budding pseudovirions incorporate the indicated orthopoxvirus membrane proteins as entry determinants, generating single-round infectious particles for entry assays. (B) Dose response inhibition of pseudovirus entry by bLf in Huh-7 cells. Huh-7 cells were infected with VSV pseudotypes bearing MPXV or VACV membrane proteins, and infection was quantified by firefly luciferase activity. Relative titers were normalized to the mean signal of vehicle-treated controls (2% FBS DMEM). Data are presented as mean ± SD from three independent experiments. (C) The impact of bLf on VSV replication was assessed using a VSV genome replication readout. Briefly, Huh-7 cells were infected with VSV G*ΔG for 2 h, followed by treatment with bovine lactoferrin for 24 h. Firefly luciferase activity was then measured as described above and used for quantification. Data are shown as mean±SD (n = 3).

**
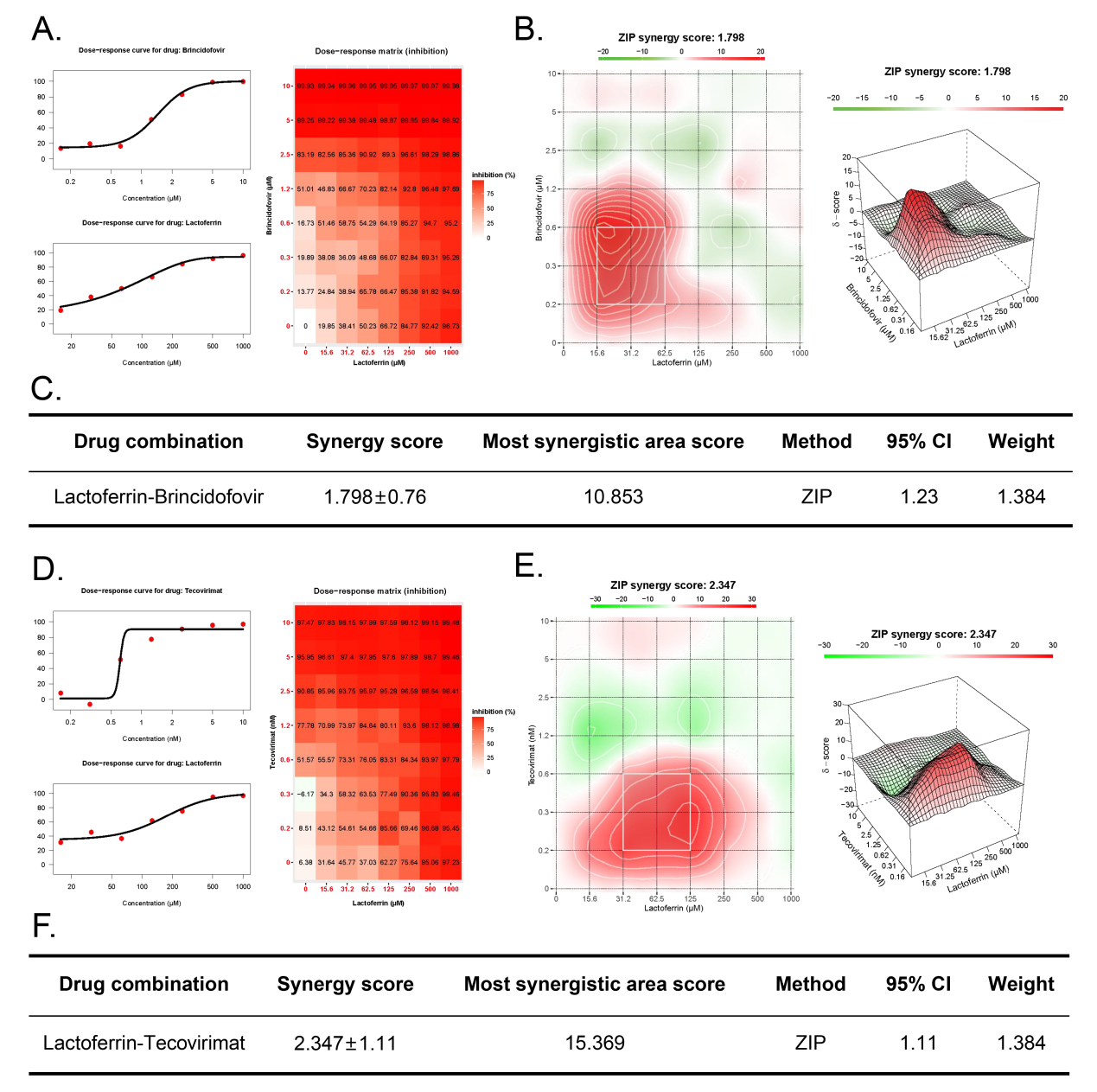
**

**Figure S5. Analysis of drug combinations on anti-vvTT activity, cell viability, and synergistic effects.** (A) Dose-response curves of lactoferrin and brincidofovir against vvTT (MOI=0.01, 36 hpi) in Vero E6 cells using 7-point, 1:2 dilution series (n=3). Heatmap shows the two-dimensional dose matrix and corresponding viral inhibition. (B) Synergy landscapes for the bLf–brincidofovir combination generated using the ZIP model in SynergyFinder 3.0. Two-dimensional (left) and three-dimensional (right) synergy maps highlight synergistic (red) and antagonistic (green) dose regions. White box indicates the dose range with the highest synergy score. (C) Summary synergy score for the lactoferrin-brincidofovir combination. SynergyFinder calculates synergy metrics with 95% CI. Scoring criteria: < -10 antagonistic; -10 to 10 additive; >10 synergistic. (D) Dose-response curves of bLf and tecovirimat against vvTT (MOI=0.01, 36 hpi) in Vero E6 cells (n=3). (E) ZIP synergy landscapes of the bLf–tecovirimat combination, displayed as 2D and 3D synergy maps. (F) Summary synergy score for the bLf–tecovirimat combination based on SynergyFinder calculations with 95% CI.

**
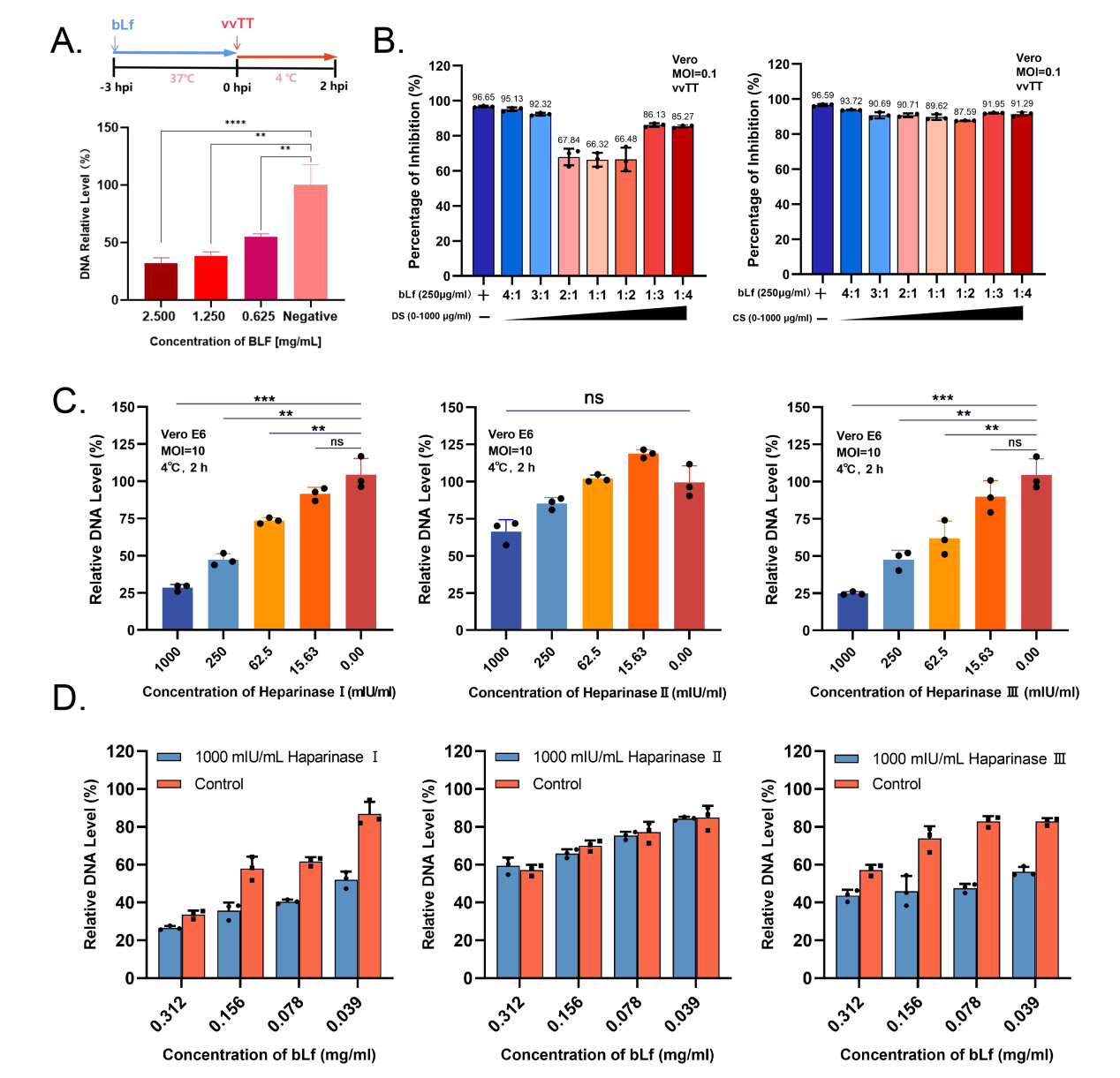
**

**Figure S6. The binding phase and function of lactoferrin. (A)** The receptor combination experiment. Vero E6 cells were pre-treated with bLf (2.5 mg/ml, 1.25 mg/ml, and 0.625 mg/ml) at 37°C for 3 h. Remove the incubation by three times PBS washing and infect cells with vvTT (MOI=10) at 37°C for 2 h. Cell-surface–adsorbed viral DNA was quantified by RT-qPCR. (B) Glycosaminoglycan competition assay. bLf (250 μg/ml) was pre-incubated with chondroitin sulfate (CS) or dermatan sulfate (DS) at indicated ratios and added during vvTT infection (MOI=0.1). Viral DNA was quantified by RT-qPCR at 24 hpi. Data = mean ± SD (n=3). (C) Heparan sulfate depletion by heparinase reduces vvTT infection. Vero E6 cells were treated with heparinase I/II/III (1000, 250, 62.5, 15.62 mIU/ml) at 37°C for 2 h, washed, and infected with vvTT (MOI=10) at 37°C for 2 h. Viral yield was quantified by RT-qPCR. Data = mean ± SEM (n=3). ****p<0.0001, ***p<0.001, **p<0.01 (one-way ANOVA with Dunnett's test). (D) Combined heparinase treatment and bLf inhibition. Vero E6 cells were pre-treated with heparinase I/II/III (1000 mIU/ml, 37°C, 2 h), washed, and then infected with vvTT (MOI=10, 2 h) in the presence of indicated bLf concentrations. Viral DNA was quantified by RT-qPCR (n=3).

**
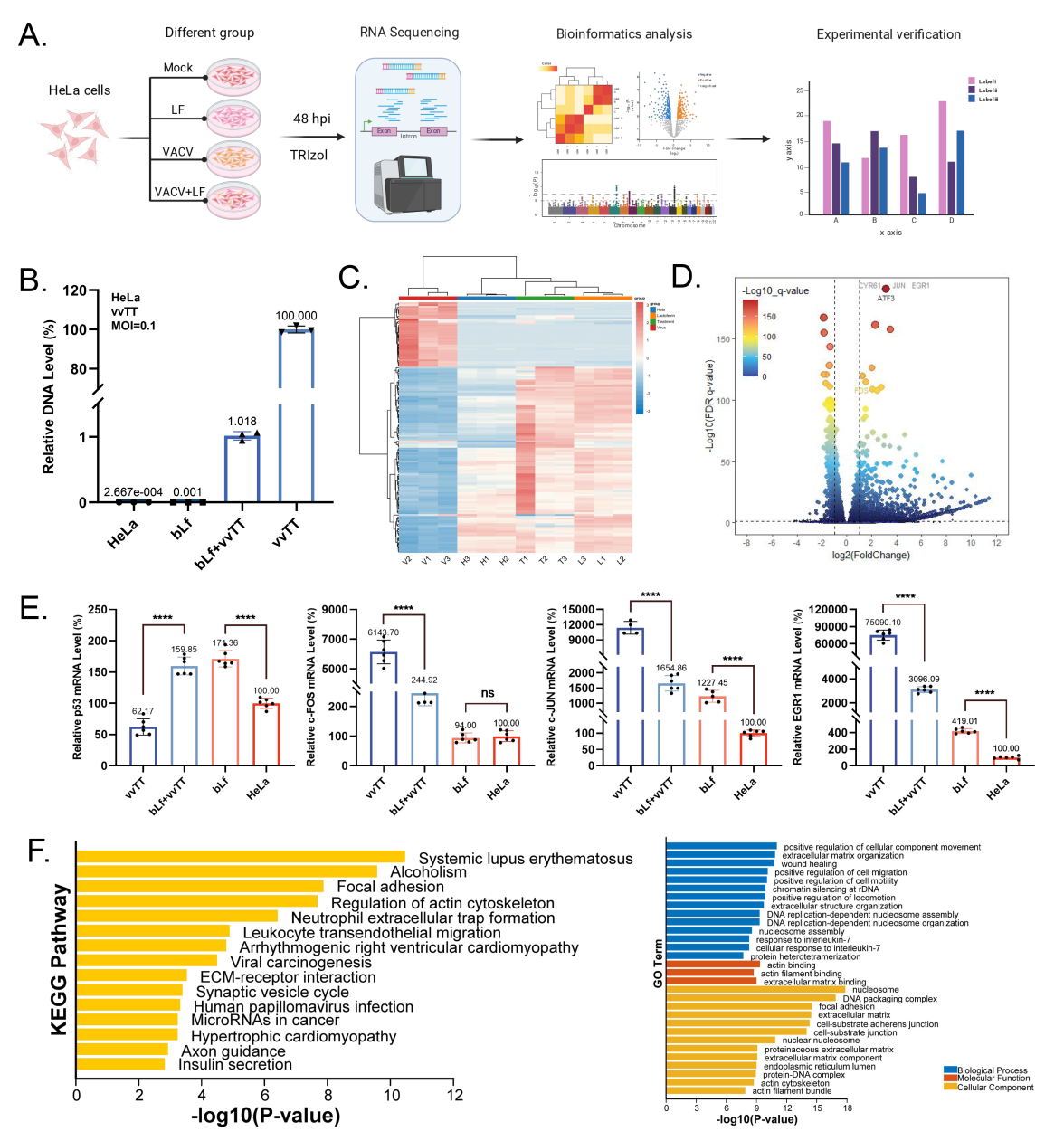
**

**Figure S7. Transcriptomic profiling reveals lactoferrin-mediated normalization of VACV-induced host immune dysregulation.** (A) Experimental design for RNA-sequencing analysis. Three biological replicates were prepared for each of four experimental groups: (i) untreated HeLa cells cultured for 48 hours; (ii) HeLa cells treated with bLf (5 mg/ml); (iii) HeLa cells infected with vvTT (MOI = 0.01); (iv) HeLa cells treated with bLf and infected with vvTT. All groups were cultured for 48 hours. Total RNA was extracted using Trizol and sequenced using standard Illumina protocols. (B) Quantitative validation of viral infection efficiency and lactoferrin antiviral activity in sequencing samples. Viral DNA quantification confirmed robust VACV replication in infected cells and significant viral suppression following bLf treatment. Data = mean ± SD (n=3). (C) Heatmap visualization of global gene expression changes across experimental groups. Red indicates upregulated genes; blue indicates downregulated genes. (D) Volcano plot displaying differentially expressed genes following vvTT infection. Red dots represent upregulated genes; blue dots represent downregulated genes, demonstrating large-scale gene expression alterations in response to viral infection. (E) qPCR validation of key transcription factors (p53, c-FOS, c-JUN, EGR1) across experimental groups. Data = mean ±SD (n=3).****p<0.0001, (two-tailed t-test). (F) Pathway enrichment analysis of differentially expressed genes in bLf-treated infected cells.

**
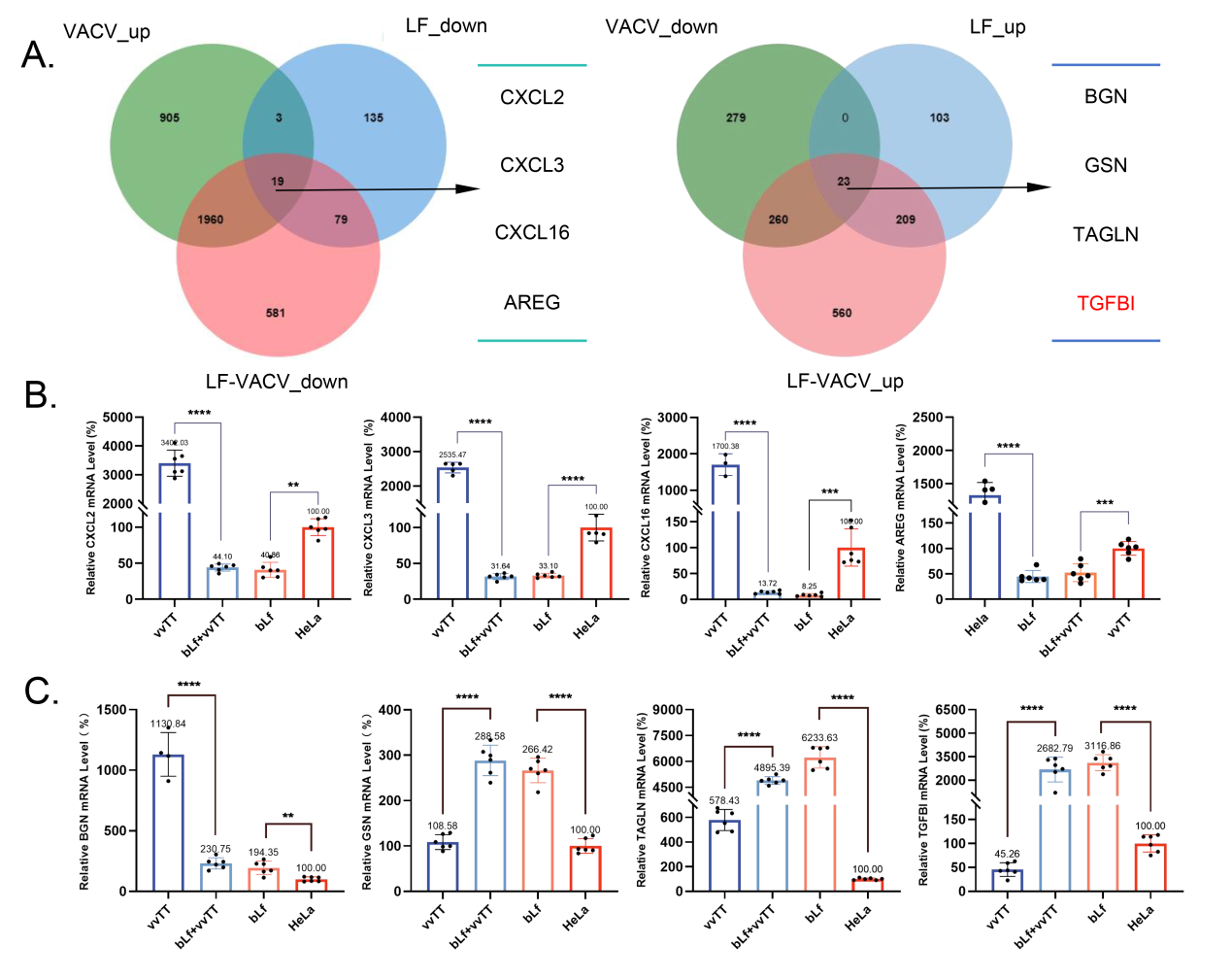
**

**Figure S8. Lactoferrin reverses VACV-induced gene expression dysregulation and activates TGFBI-mediated antiviral signaling pathways. (A)** Venn diagram showing gene overlaps with contrasting regulatory patterns between VACV infection and bLf treatment. Screening revealed 19 and 23 overlapping genes in respective comparisons. **(B)** RT-qPCR validation of lactoferrin-downregulated chemokine genes (CXCL2, CXCL3, CXCL16) and epidermal growth factors (AREG) in mock-infected and VACV-infected cells. Lactoferrin treatment reversed VACV-induced upregulation of these pro-inflammatory chemokines across biological process and molecular function categories. Data = mean ± SD (n=3), ****p <0.0001, ***p <0.001, **p <0.01 (two-tailed t-test). **(C)** RT-qPCR quantification of lactoferrin-upregulated genes (BGN, GSN, TAGLN, TGFBI). Lactoferrin treatment upregulated BGN, GSN, TAGLN, and TGFBI. Data = mean ± SD (n=3), ****p <0.0001, **p <0.01 (two-tailed t-test).

**
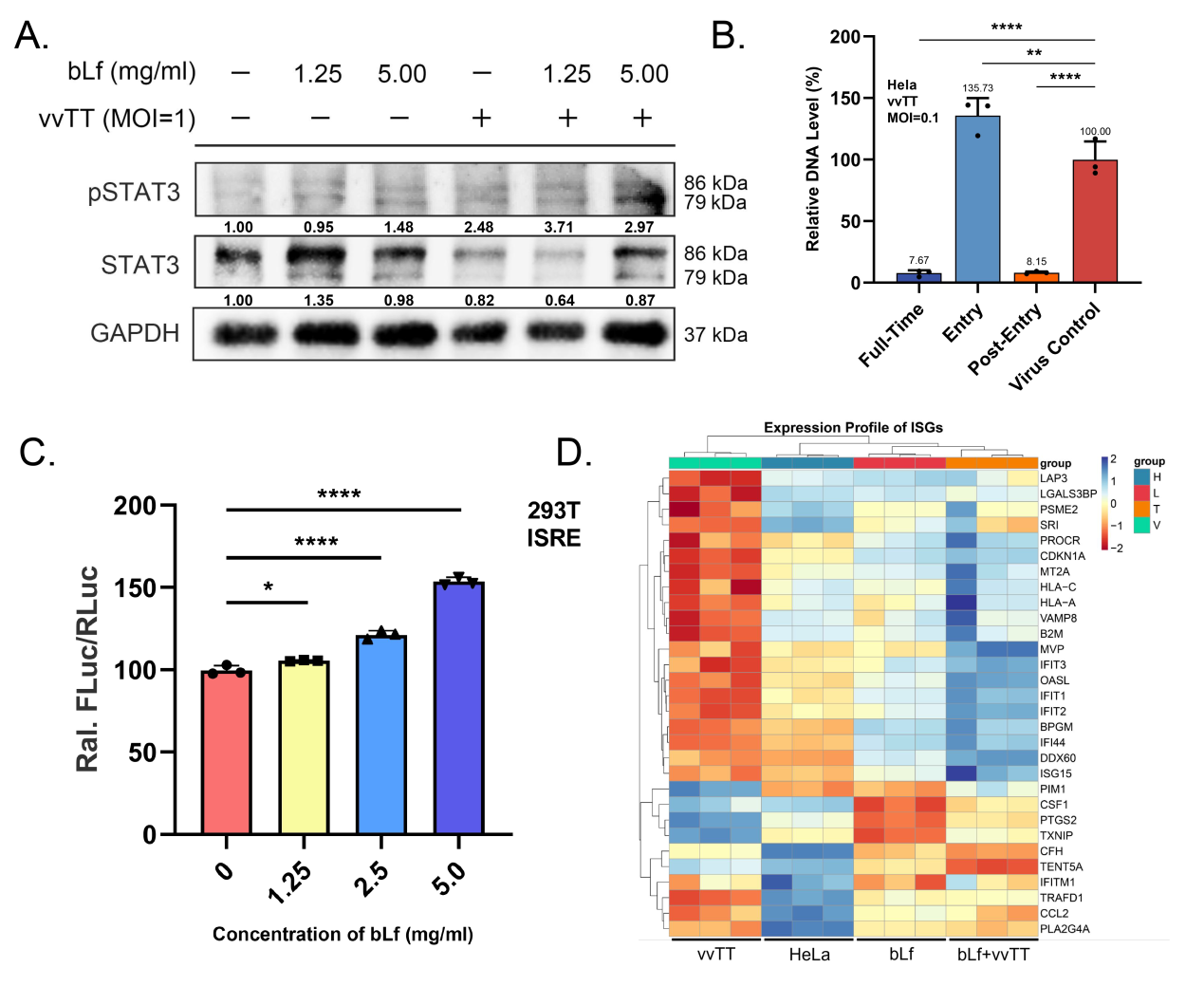
Figure S9. Lactoferrin activates innate immune pathways to exert antiviral effects. (A)** Western blot analysis of STAT3 and phospho-STAT3 (pSTAT3) expression in HeLa cells under indicated treatment conditions. GAPDH served as loading control. Band intensities were quantified using ImageJ. Representative blot and quantification from three independent experiments are shown. **(B)** Time-of-addition assay with 400 nM anisomycin against vvTT infection (MOI=0.1) in HeLa cells. Full-Time: present throughout infection; Entry: during 2 h adsorption only; Post-Entry: added at 2 hpi. Viral DNA quantified by RT-qPCR at 14 hpi. Data = mean ± SD (n=3). ***p<0.001, **p<0.01 vs. Virus control (one-way ANOVA with Dunnett's test). **(C)** Dose-dependent activation of interferon-stimulated response element (ISRE) promoter activity by lactoferrin. 293T cells were transfected with ISRE-luciferase reporter and treated with lactoferrin at concentrations of 1.25, 2.5, and 5.0 mg/ml for 24 hours without viral infection. Luciferase activity was measured by dual-luciferase assay and normalized to Renilla control. Lactoferrin dose-dependently induced ISRE promoter activity, confirming activation of interferon-mediated antiviral responses. Data = mean ± SD (n=3). **(D) Heatmap of ISGs from MSigDB gene sets. Color scale represents normalized expression (z-score).**

**Table S1.** Sequences of primer in this paper.

| Oligonucleotide name | Sequence (5′-3′) |  |
| --- | --- | --- |
| VACV-F | TGCTTGGTATAAGGAGCCCA | |
| VACV-R | GTACCGGCATCTCTAGCAGTC | |
| Human GAPDH-F | AGCCTCAAGATCATCAGCAATG | |
| Human GAPDH-R | ATGGACTGTGGTCATGAGTCCTT | |
| CXCL2-F | GCTTGTC TCAACCCCGCATC | |
| CXCL2-R | TGGATTTGCCATTTTTCAGCATCTT | |
| CXCL3-F | GCAGGGAATTCACCTCAAGA | |
| CXCL3-R | TTTTCGATGATTTTCTGAACCA | |
| CXCL16-F | CAGTTTCAGAGCACCCAGCAGTC | |
| CXCL16-R | GCCTAGCCTCCAGACCATAGCC | |
| AREG-F | GCACCTGGAAGCAGTAACATGC | |
| AREG-R | GGCAGCTATGGCTGCTAATGCA | |
| BGN-F | AATGAACTCCACCTAGACCACAA | |
| BGN-R | GATGTTGTTGGAGTGCGATAGAC | |
| GSN-F | TGGGAGAGCTTCAACAATGGC | |
| GSN-R | ACCACTGGTGGATGTTGTTGC | |
| TAGLN-F | TCCAGGTCTGGCTGAAGAATGG | |
| TAGLN-R | CTGCTCCATCTGCTTGAAGACC | |
| TGFBⅠ-F | TACCTGAACCCGTGTTGCTCTC | |
| TGFBⅠ-R | GTTGCTGAGGTATCGCCAGGAA | |
| p53-F | CCTCAGCATCTTATCCGAGTGG | |
| p53-R | TGGATGGTGGTACAGTCAGAGC | |
| c-FOS-F | GCCTCTCTTACTACCACTCACC | |
| c-FOS-R | AGATGGCAGTGACCGTGGGAAT | |
| c-JUN-F | CAGTCCAGCAATGGGCACATCA | |
| c-JUN-R | GGAAGCGTGTTCTGGCTATGCA | |
| EGR1-F | AGCAGCACCTTCAACCCTCAGG | |
| EGR1-R | GAGTGGTTTGGCTGGGGTAACT | |
| IFN-α-F | AGAAGGCTCCAGCCATCTCTGT | |
| IFN-α-R | TGCTGGTAGAGTTCGGTGCAGA | |
| IFN-β-F | CTTGGATTCCTACAAAGAAGCAGC | |
| IFN-β-R | GGAAGACAGGAGAGCTGCAACT | |
| IFN-λ-F | AACTGGGAAGGGCTGCCACATT | |
| IFN-λ-R | GGAAGACAGGAGAGCTGCAACT | |
